# Bis-Cyclometalated Indazole and Benzimidazole Chiral-at-Iridium Complexes: Synthesis and Asymmetric Catalysis

**DOI:** 10.3390/molecules26071822

**Published:** 2021-03-24

**Authors:** Sebastian Brunen, Yvonne Grell, Philipp S. Steinlandt, Klaus Harms, Eric Meggers

**Affiliations:** Fachbereich Chemie, Philipps-Universität Marburg, Hans-Meerwein-Strasse 4, 35043 Marburg, Germany; sbrunen@kofo.mpg.de (S.B.); Grelly@staff.uni-marburg.de (Y.G.); philipp.steinlandt@googlemail.com (P.S.S.); xray15@posteo.de (K.H.)

**Keywords:** cyclometalation, chiral-at-metal, asymmetric catalysis

## Abstract

A new class of bis-cyclometalated iridium(III) catalysts containing two inert cyclometalated 6-*tert*-butyl-2-phenyl-2*H*-indazole bidentate ligands or two inert cyclometalated 5-*tert*-butyl-1-methyl-2-phenylbenzimidazoles is introduced. The coordination sphere is complemented by two labile acetonitriles, and a hexafluorophosphate ion serves as a counterion for the monocationic complexes. Single enantiomers of the chiral-at-iridium complexes (>99% er) are obtained through a chiral-auxiliary-mediated approach using a monofluorinated salicyloxazoline and are investigated as catalysts in the enantioselective conjugate addition of indole to an α,β-unsaturated 2-acyl imidazole and an asymmetric Nazarov cyclization.

## 1. Introduction

**Figure 1:**
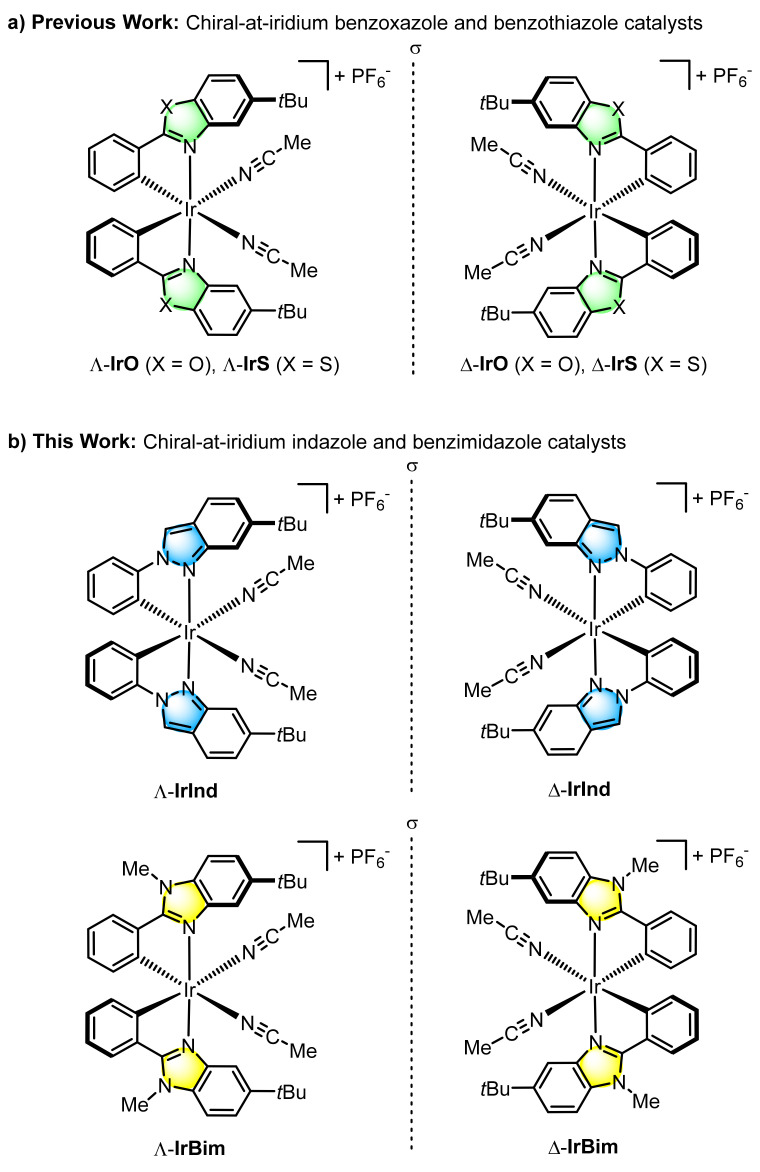
Bis-cyclometalated chiral-at-iridium catalysts for asymmetric conversions. (**a**) Our most successful previous design. (**b**) This study.

Cyclometalated transition metal complexes are not only of tremendous importance as intermediates in C–H functionalization reactions but have also gained increasing attention for their interesting catalytic and biological properties, not least due to the pioneering contributions of the Pfeffer laboratory on the formation and applications of metallacycles [1,2,3,4]. Our group has recently introduced bis-cyclometalated iridium and rhodium complexes as chiral catalysts for application in asymmetric catalysis [5,6]. Our initial design was based on iridium(III) complexes in which two inert cyclometalated 5-*tert*-butyl-2-phenylbenzoxazoles (**IrO [7]**) or 5-*tert*-butyl-2-phenylbenzothiazoles (**IrS [8]**) are complemented by two labile acetonitrile molecules and hexafluorophosphate served as the counterion for the monocationic complexes (Figure 1a). The two cyclometalated ligands implement a stereogenic metal center with either a left-handed (Λ-configuration) or right-handed (Δ-configuration) overall helical topology [9,10,11,12,13,14,15,16,17,18,19,20,21]. The strongly σ-donating phenyl ligands labilize the acetonitrile ligands by exerting a strong *trans* effect [22]. This is important for catalysis, during which one or two acetonitrile ligands are replaced by a substrate or reagent, while the helically arranged inert cyclometalated ligands provide the asymmetric induction. 

Since the nature of the cyclometalating ligands affects the reactivity and stereoselectivity of the cyclometalated complexes during catalysis, as has been already witnessed in the differences between benzoxazole and benzothiazole catalysts [5], we aimed to investigate related complexes in which the benzoxazole or benzothiazole heterocycles were replaced with different heterocyclic ligands [23]. Here, we report our results on two new classes of bis-cyclometalated chiral-at-iridium catalysts, which are based on cyclometalated 6-*tert*-butyl-2-phenyl-2*H*-indazoles (Λ- and Δ-**IrInd**) or 5-*tert*-butyl-1-methyl-2-phenylbenzimidazoles (Λ- and Δ-**IrBim**) (Figure 1b). We disclose the synthesis of enantiomerically pure complexes and provide initial results of catalytic asymmetric transformations.

## 2. Results and Discussion

**Scheme 1:**
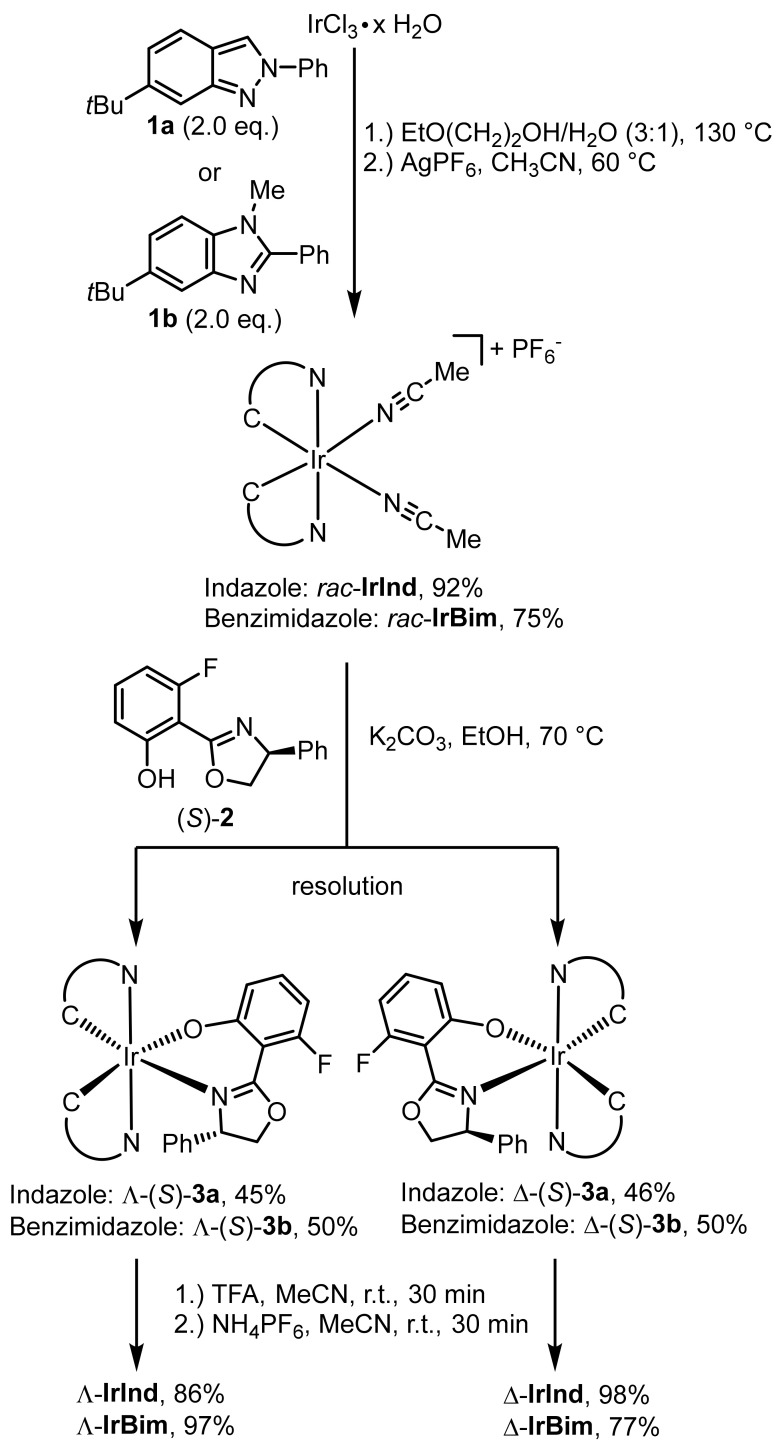
Auxiliary-mediated synthesis of enantiopure Λ- and Δ-**IrInd,** and Λ- and Δ-**IrBim**.

**Figure 2:**
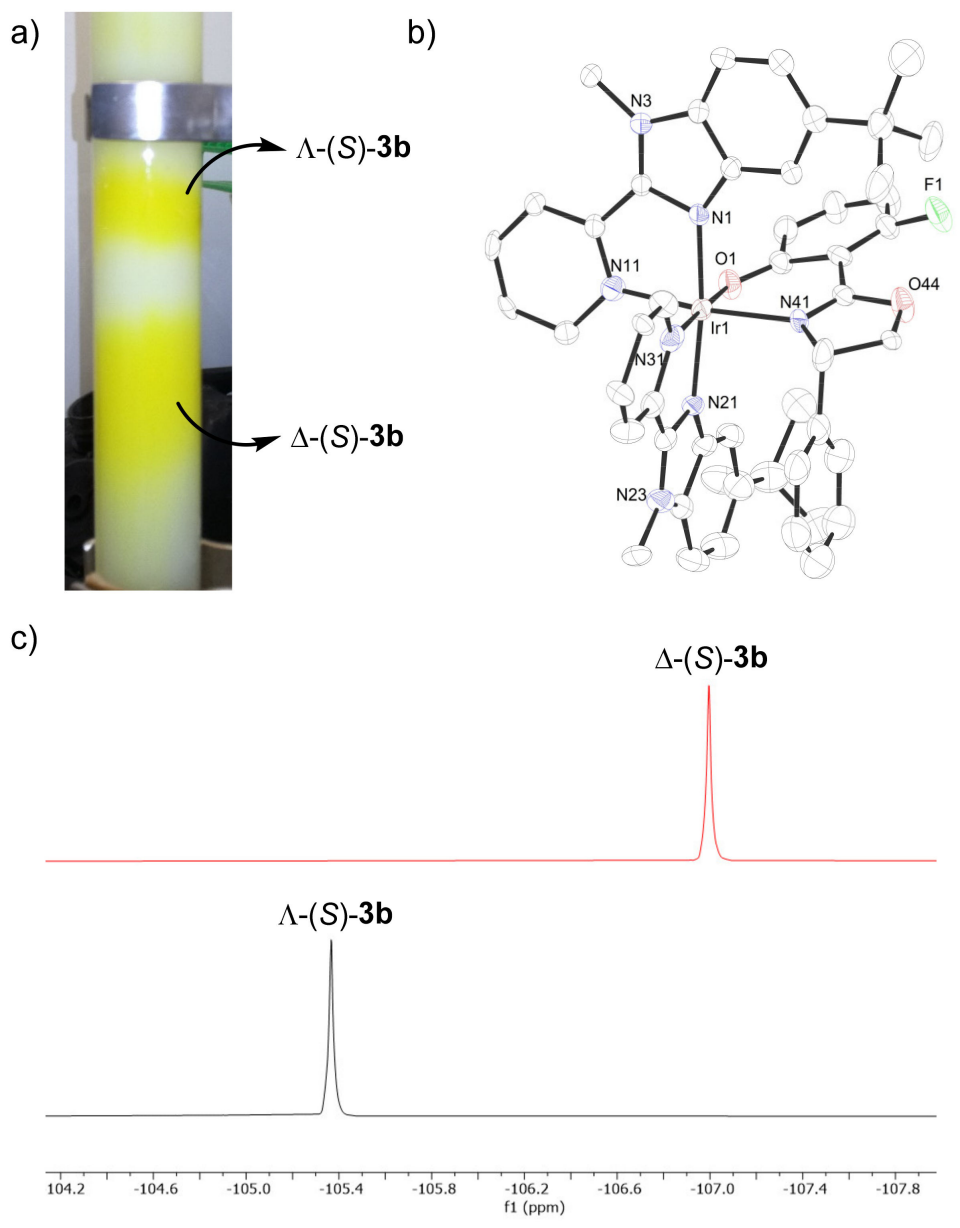
Auxiliary complexes of the iridium benzimidazole system. **a**) Chromatographic separation of Λ-(*S*)-**3b** and Δ-(*S*)-**3b** during silica gel column chromatography (*n*-pentane/EtOAc). **b**) Single-crystal X-ray structure of Λ-(*S*)-**3b**. ORTEP drawing with 30% probability thermal ellipsoids. Solvent molecules are omitted for clarity. **c**) ^19^F-NMR spectra of the individual diastereomers Λ-(*S*)-**3b** and Δ-(*S*)-**3b**.

**Figure 3:**
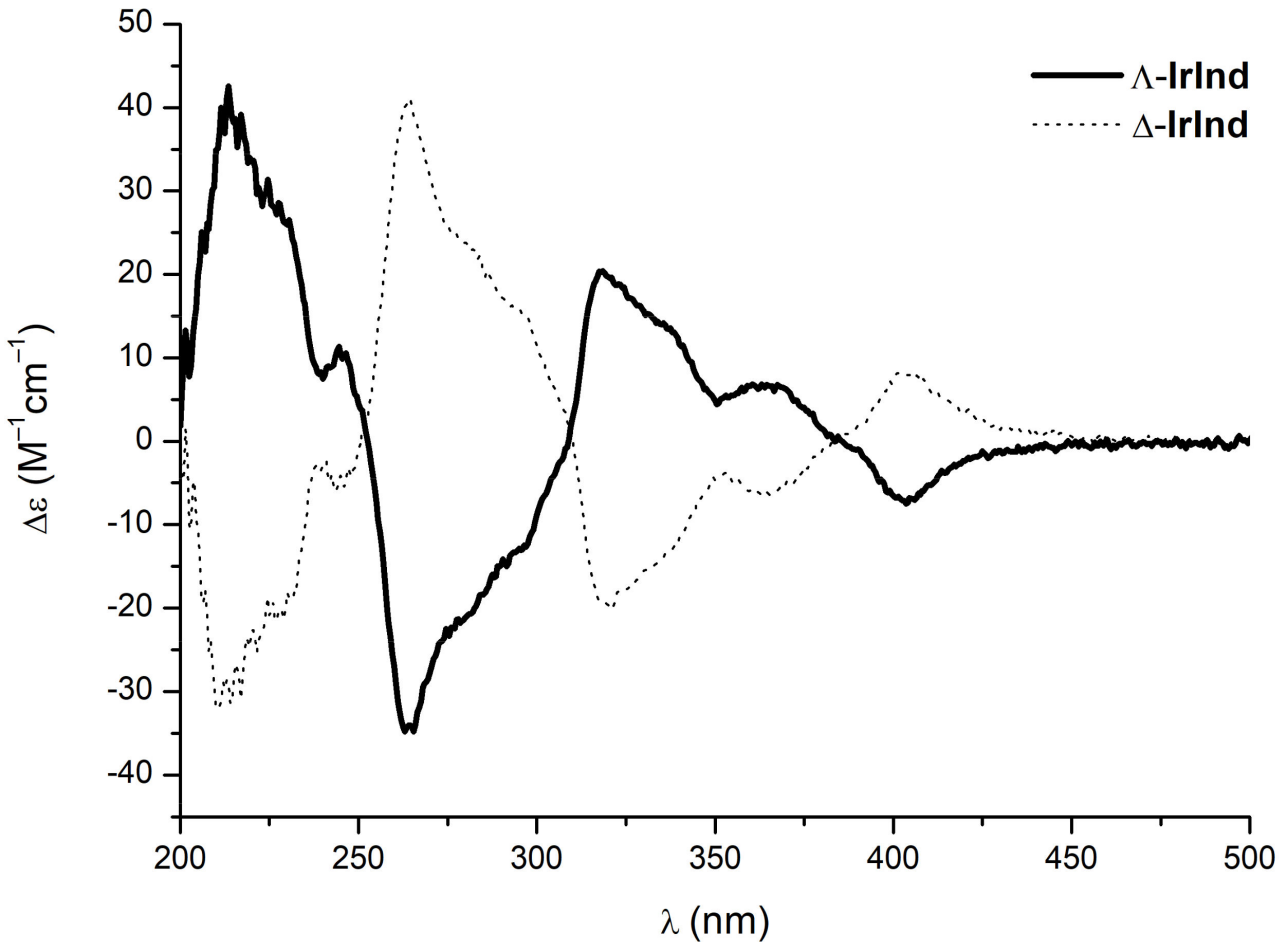
CD spectra of Λ- and Δ-**IrInd** in MeOH (0.2 mM).

**Figure 4:**
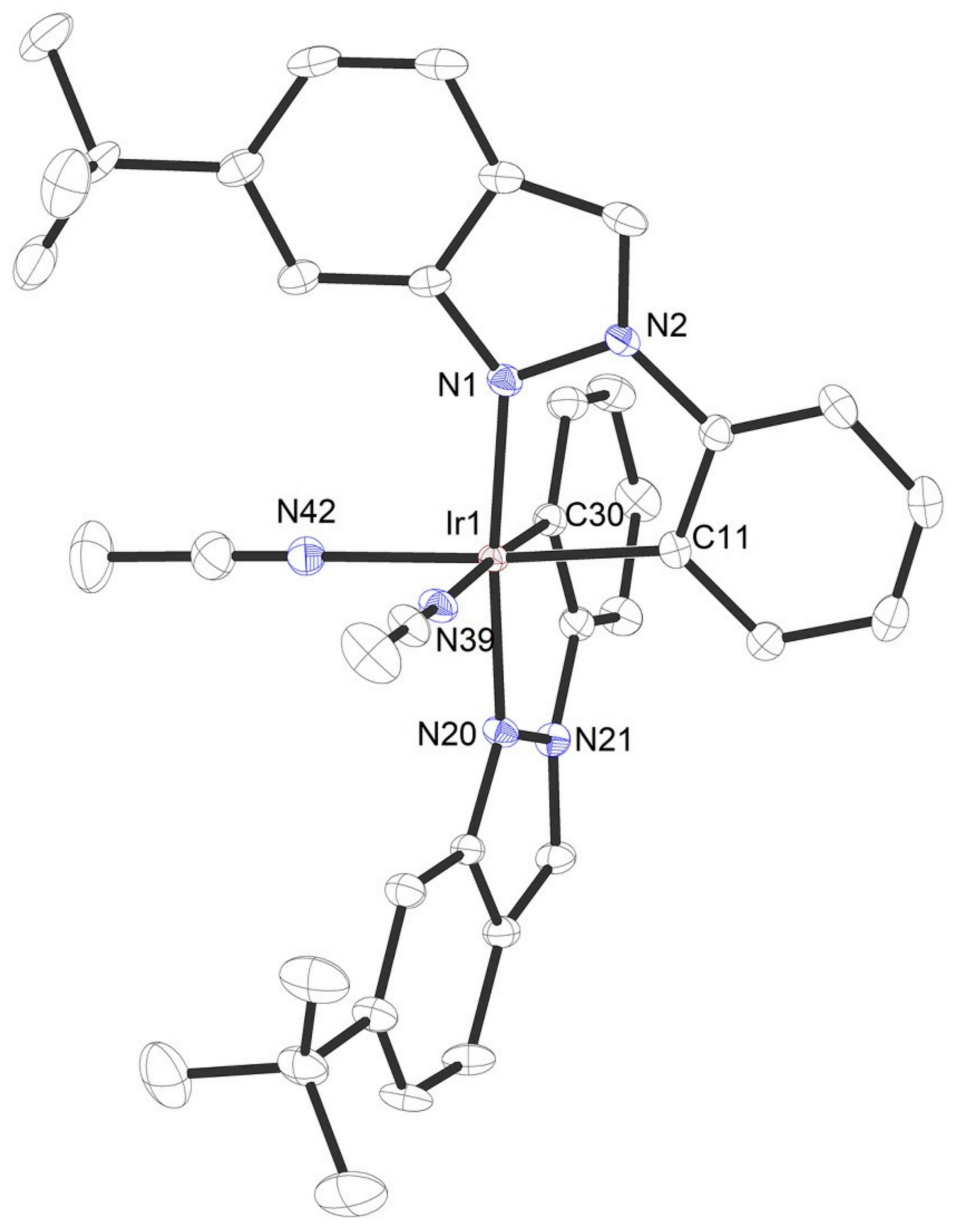
Crystal structure of Λ-**IrInd**. ORTEP drawing with 30% probability thermal ellipsoids. Hexafluorophosphate counterion and solvent molecules are omitted for clarity.

Racemic bis-cyclometalated iridium complexes with cyclometalated 2-phenyl-2*H*-indazole [24,25,26,27] or 2-phenylbenzimidazole [28,29,30,31,32] ligands are well established but the catalytic properties of non-racemic complexes have not yet been reported. The chiral-auxiliary-mediated [33,34,35,36] synthesis of enantiomerically pure complexes **IrInd** and **IrBim** was performed in analogy to **IrO [7]** and **IrS [8]** and started with the reaction of iridium chloride hydrate with two equivalents of 6-*tert*-butyl-2-phenyl-2*H*-indazole (**1a**) or 5-*tert*-butyl-1-methyl-2-phenylbenzimidazole (**1b**) in a mixture of 2-ethoxyethanol and water (3:1) at 130 °C for 24 h, followed by treatment with silver hexafluorophosphate in acetonitrile to afford *rac*-**IrInd** in 92% yield and *rac*-**IrBim** in 75% yield (Scheme 1). The smooth bis-cyclometalation to provide *rac*-**IrInd** was noteworthy and the yield was significantly higher compared to *rac*-**IrBim** and the previously reported *rac*-**IrO** and *rac*-**IrS** [7,8]. It is interesting to note that the related rhodium benzimidazole complex could not be obtained by this route and stopped after the first cyclometalation step [37,38]. Next, for obtaining single enantiomers, the racemic iridium complexes were reacted with the monofluorinated salicyloxazoline (*S*)-**2 [39,40]** in EtOH at 70 °C for 6 hours in the presence of 3.0 equivalents of K_2_CO_3_. This converted the racemic mixtures into pairs of diastereomers, which could easily be separated by standard silica gel chromatography (see Figure 2a for an example). For the indazole ligand, Λ-(*S*)-**3a** was obtained in 45% yield and Δ-(*S*)-**3a** in 46% yield, while for the benzimidazole ligand, Λ-(*S*)-**3b** and Δ-(*S*)-**3b** were both obtained in 50% yield. The relative and absolute configuration of Λ-(*S*)-**3b** was determined by single-crystal X-ray diffraction (Figure 2b), and the other complexes were assigned accordingly with the help of circular dichroism (see Supporting Information). The diastereomeric purity of the complexes was investigated by NMR spectroscopy. Figure 2c shows the ^19^F-NMR spectra of Λ-(*S*)-**3b** and Δ-(*S*)-**3b**, which demonstrate the high diastereomeric purities of the isolated auxiliary-coordinated complexes. Such high diastereomeric purities are crucial for later obtaining enantiomerically pure catalysts. Finally, cleavage of the auxiliary was conducted with trifluoroacetic acid (TFA) (6.0 equivalents) in MeCN for 30 min at room temperature, and subsequent treatment with NH_4_PF_6_ provided the individual non-racemic complexes Λ-**IrInd** (86%), Δ-**IrInd** (98%), Λ-**IrBim** (97%), and Δ-**IrBim** (77%) as hexafluorophosphate salts. HPLC analysis on a chiral stationary phase revealed that all four complexes were virtually enantiomerically pure, with enantiomeric ratios of larger than 99:1 (see Supporting Information for HPLC traces). The CD spectra in Figure 3 for Λ- and Δ-**IrInd** demonstrate the mirror-image character of the isolated complexes. The assigned absolute configurations were also confirmed with a crystal structure of Λ-**IrInd** (Figure 4). 

Next, we investigated the catalytic properties of the new chiral-at-iridium complexes. Over the past several years, we [5] and others [41,42,43] have demonstrated that bis-cyclometalated iridium(III) complexes are versatile chiral Lewis acid catalysts. To compare the catalytic activity of the new indazole complex **IrInd** and the benzimidazole complex **IrBim** with the established benzoxazole catalyst **IrO [7]** and benzothiazole catalyst **IrS** [8,44], we chose the enantioselective Friedel–Crafts alkylation of indole with the α,β-unsaturated 2-acyl imidazole **4** (Table 1) [45,46,47,48]. At a catalyst loading of 2.0 mol%, Λ-**IrInd** afforded (*S*)-**5** in 84% yield and with 98.5% ee, while Δ-**IrBim** (2.0 mol%) provided (*R*)-**5** in a lower yield of 81% and with slightly decreased 98% ee. In this reaction, **IrBim** also showed lower catalytic activity and required a reaction time of 49 hours, compared to 29 hours for **IrInd**, to complete the reaction at room temperature. However, both catalysts displayed markedly lower catalytic activities compared to the benzoxazole **IrO** and the benzothiazole **IrS**, which could be used at the reduced catalyst loading of just 1.0 mol%. Furthermore, Λ-**IrS** provided (*S*)-**5** with a superior enantiomeric excess of 99% ee [44].

As a second model reaction, we chose an asymmetric Nazarov cyclization [49,50]. We recently demonstrated that Λ-**IrS** (2.0 mol%) is an excellent chiral Lewis acid for the conversion of ketoester **6** to the cyclopentenone (1*R*,2*S*)-**7** (Table 2) [51]. Using the same established reaction conditions, after a reaction time of 7 hours in hexafluoroisopropanol [52] at 50 °C, followed by base-induced isomerization to the thermodynamic *trans* diastereomer, (1*R*,2*S*)-**7** was obtained in 75% yield with 15:1 dr and 93% ee. For comparison, **IrInd** and **IrBim** displayed somewhat lower catalytic activity and required an elongated reaction time of 24 hours but, at the same time, afforded higher enantioselectivities compared to **IrS**. Accordingly, Λ-**IrInd** (2.0 mol%) provided (1*R*,2*S*)-**7** in a moderate 46% yield with 11:1 dr and 96% ee, while Δ-**IrBim** (2.0 mol%) provided (1*S*,2*R*)-**7** in 73% yield with 12.5:1 dr and 94% ee. 

## 3. Materials and Methods

All reactions were carried out under an atmosphere of nitrogen with magnetic stirring in flame-dried glassware, unless stated otherwise. Catalytic reactions were performed in Schlenk tubes (10 mL). Solvents were distilled under nitrogen from sodium/benzophenone (THF, Et_2_O) or calcium hydride (MeCN, CH_2_Cl_2_, CHCl_3_, toluene). Commercially purchased compounds were used without further purification. Flash column chromatography was performed with silica gel 60 M from Macherey-Nagel (irregular shaped, 230–400 mesh, pH 6.8, pore volume: 0.81 mL g^−1^, mean pore size: 66 A, specific surface: 492 m^2^ g^−1^, particle size distribution: 0.5% < 25 μm and 1.7% > 71 μm, water content: 1.6%). ^1^H NMR, ^13^C{^1^H} NMR, and ^19^F{^1^H, ^13^C} NMR spectra were recorded on a Bruker AV II 300-MHz, AV III HD 250-MHz, AVIII 500-MHz, AV III HD 500-MHz, or AV II 600-MHz spectrometer at room temperature. The chemical shift δ is listed in ppm with the solvent resonance as internal standard. High-resolution mass spectrometry was conducted via the electrospray ionization technique (ESI) or atmospheric pressure chemical ionization technique (APCI) on a Finnigan LTQ-FT Ultra mass spectrometer (Thermo Fischer Scientific, Bremen, Germany). CD spectra were recorded on a JASCO J-810 CD spectropolarimeter (parameters: path length of cuvette 1.0 mm, bandwidth 1 nm, data pitch 0.5 nm, response 1 second, sensitivity standard, scanning speed 50 nm/min, accumulation of 3 scans).

***rac*-IrInd.** Iridium(III) chloride hydrate (w_Ir_ = 53%, 350 mg, 0.97 mmol, 1.00 equiv.) and 6-(*tert*-butyl)-2-phenyl-2*H*-indazole (483 mg, 1.93 mmol, 2.00 equiv.) were added to a Schlenk flask, a mixture of 2-ethoxyethanol and H_2_O (*v*/*v* = 3:1, 40 mL, 0.025 M) was added and the resulting suspension was heated to 130 °C for 24 h. The mixture was cooled to room temperature, concentrated under reduced pressure and the obtained residue was dried under vacuum. AgPF_6_ (736 mg, 2.91 mmol, 3.00 equiv.) and MeCN (25 mL, 0.04 M) were added, and the resulting suspension was heated to 60 °C for 14 h. The obtained suspension was cooled to room temperature, filtered through a short plug of Celite, rinsed with MeCN and concentrated under reduced pressure. The crude product was purified via silica gel column chromatography (CH_2_Cl_2_/MeCN 20:1 → 10:1) to yield *rac*-**IrInd** (811 mg, 0.88 mmol, 92%) as a yellow solid. TLC: R*_f_* = 0.48 (CH_2_Cl_2_/MeCN 10:1). ^1^H-NMR: 300 MHz, CD_2_Cl_2_; *δ*/ppm = 8.68 (s, 2 H, H_arom._), 7.85 (d, ^3^*J* = 9.4 Hz, 4 H, H_arom._), 7.50 (dd, ^3^*J* = 8.8 Hz, ^4^*J* = 1.2 Hz, 2H, H_arom._), 7.47 (d, ^3^*J* = 7.9 Hz, 2H, H_arom._), 6.97 (t, ^3^*J* = 7.8 Hz, 2H, H_arom._), 6.64 (t, ^3^*J* = 7.0 Hz, 2H, H_arom._), 5.88 (d, ^3^*J* = 7.3 Hz, 2H, H_arom._), 2.35 (s, 6H, H_MeCN_), 1.45 (s, 18H, H_2x*t*-butyl_). ^13^C-NMR: 75 MHz, CD_2_Cl_2_; *δ*/ppm = 154.9 (2C), 148.8 (2C), 143.4 (2C), 134.3 (2C), 127.7 (2C), 127.6 (2C), 124.1 (2C), 124.0 (2C), 122.0 (2C), 121.6 (2C), 120.8 (2C), 120.3 (2C), 113.3 (2C), 109.3 (2C), 36.1 (2C), 31.3 (6C), 4.1 (2C). ^19^F-NMR: 282 MHz, CD_2_Cl_2_; *δ*/ppm = −72.67 (d, *J*_P-F_ = 712.6 Hz, 6F). HRMS: (ESI+, *m*/*z*) calc. for C_38_H_40_IrN_6_ [M-PF_6_]: 773.2944, found: 773.2940.

***rac*-IrBim.** Iridium(III) chloride hydrate (w_Ir_ = 53%, 400 mg, 1.10 mmol, 1.00 equiv.) and 5-(*tert*-butyl)-1-methyl-2-phenyl-1*H*-benzo[*d*]imidazole (583 mg, 2.21 mmol, 2.00 equiv.) were added to a Schlenk flask, a mixture of 2-ethoxyethanol and H_2_O (*v*/*v* = 3:1, 44 mL, 0.025 M) was added and the resulting suspension was heated to 130 °C for 24 h. The mixture was cooled to room temperature, concentrated under reduced pressure and the obtained residue was dried under vacuum. AgPF_6_ (832 mg, 3.30 mmol, 3.00 equiv.) and MeCN (27 mL, 0.04 M) were added and the resulting suspension was heated to 60 °C for 14 h. The obtained suspension was cooled to room temperature, filtered through a short plug of Celite, rinsed with MeCN and concentrated under reduced pressure. The crude product was purified via silica gel column chromatography (CH_2_Cl_2_/MeCN 20:1 → 10:1) to yield *rac*-**IrBim** (776 mg, 0.82 mmol, 75%) as yellow solid. TLC: R*_f_* = 0.43 (CH_2_Cl_2_/MeCN 10:1). ^1^H-NMR: 300 MHz, CD_2_Cl_2_; *δ*/ppm = 7.93 (d, ^4^*J* = 1.3 Hz, 2H, H_arom._), 7.76 (dd, ^3^*J* = 8.0 Hz, ^4^*J* = 0.7 Hz, 2H, H_arom.)_, 7.65 (dd, ^3^*J* = 8.8 Hz, ^4^*J* = 1.7 Hz, 2H, H_arom._), 7.58 (d, ^3^*J* = 8.7 Hz, 2 H, H_arom._), 6.96 (dt, ^3^*J* = 7.5 Hz, ^4^*J* = 1.1 Hz, 2H, H_arom._), 6.69 (dt, ^3^*J* = 7.5 Hz, ^4^*J* = 1.1 Hz, 2H, H_arom._), 6.19 (dd, ^3^*J* = 7.8 Hz, ^4^*J* = 0.7 Hz, 2H, H_arom._), 4.32 (s, 6H, H_methyl_), 2.34 (s, 6H, H_MeCN_), 1.46 (s, 18 H, H_2x*t*-butyl_). ^13^C-NMR: 75 MHz, CD_2_Cl_2_; *δ*/ppm = 163.1 (2C), 148.6 (2C), 144.9 (2C), 140.4 (2C), 135.3 (2C), 134.2 (2C), 133.8 (2C), 130.0 (2C), 124.9 (2C), 123.0 (2C), 122.9 (2C), 119.9 (2C), 112.5 (2C), 110.6 (2C), 35.5 (2C), 32.8 (2C), 32.1 (6C), 4.2 (2C). ^19^F-NMR: 282 MHz, CD_2_Cl_2_; *δ*/ppm = −72.99 (d, *J*_P-F_ = 710.7 Hz, 6F). HRMS: (ESI+, *m*/*z*) calc. for C_40_H_44_IrN_6_ [M-PF_6_]: 801.3257, found: 801.3253.

**Λ-(*S*)-3a andΔ-(*S*)-3a.***rac*-**IrInd** (250 mg, 0.27 mmol, 1.00 equiv.), (*S*)-**2** (77.1 mg, 0.30 mmol, 1.10 equiv.), and K_2_CO_3_ (0.81 mmol, 112 mg, 3.00 equiv.) were added to a Schlenk tube, the tube was evacuated for 5 min and absolute ethanol (11 mL, 0.025 M) was added. The tube was sealed and heated to 70 °C for 6 h under a nitrogen atmosphere. The mixture was diluted with CH_2_Cl_2_ (10 mL) and filtered through a short plug of Celite. The filtrate was concentrated under reduced pressure and the crude product was purified by silica gel column chromatography (*n*-pentane/EtOAc 20:1 → 2:1) to yield Λ-(*S*)-**3a** (115 mg, 121 μmol, 45%) and Δ-(*S*)-**3a** (118 mg, 124 μmol, 46%), both as brown solids.

Analytical data for Λ-(*S*)-**3a**: TLC: R*_f_* = 0.77 (*n*-pentane/EtOAc 2:1). ^1^H-NMR: 300 MHz, CD_2_Cl_2_; *δ*/ppm = 8.51 (s, 1H, H_arom._), 8.08 (s, 1H, H_arom._), 7.77 (s, 1H, H_arom._), 7.67 (d, ^3^*J* = 9.0 Hz, 1H, H_arom._), 7.54 (s, 1H, H_arom._), 7.44 (d, ^3^*J* = 9.0 Hz, 1H, H_arom._), 7.36 (d, ^3^*J* = 7.9 Hz, 1H, H_arom._), 7.35 (dd, ^3^*J* = 9.0 Hz, ^4^*J* = 1.3 Hz, 1H, H_arom._), 7.27 (dd, ^3^*J* = 9.0 Hz, ^4^*J* = 1.4 Hz, 1H, H_arom._), 7.04 (dd, ^3^*J* = 8.0 Hz, ^4^*J* = 0.8 Hz, 1H, H_arom._), 6.99–6.91 (m, 1H, H_arom._), 6.90–6.83 (m, 2H, H_arom._), 6.78 (t, ^3^*J* = 7.4 Hz, 1H, H_arom._), 6.65–6.39 (m, 6H, H_arom._), 6.19 (d, ^3^*J* = 7.3 Hz, 2H, H_arom._), 5.95 (ddd, ^3^*J* = 12.9 Hz, ^3^*J* = 8.0 Hz, ^4^*J* = 0.9 Hz, 1H, H_arom._), 5.58 (dd, ^3^*J* = 7.6 Hz, ^4^*J* = 1.0 Hz, 1H, H_arom._), 4.92 (dd, ^3^*J* = 9.5 Hz, ^4^*J* = 4.4 Hz, 1H, H_aliph._), 4.82 (dd, ^3^*J* = 9.7 Hz, ^3^*J* = 9.6 Hz, 1H, H_aliph._), 4.01 (dd, ^3^*J* = 8.6 Hz, ^4^*J* = 4.4 Hz, 1H, H_aliph._), 1.43 (s, 9H, H*_t_*_-butyl_), 1.27 (s, 9H, H*_t_*_-butyl_). ^13^C-NMR: 75 MHz, CD_2_Cl_2_; *δ*/ppm = 173.8 (d, *J*_C-F_ = 3.3 Hz), 165.4, 163.2 (d, *J*_C-F_ = 3.6 Hz), 162.0, 153.5, 153.0, 149.0, 148.4, 145.3, 144.5, 140.7, 138.6, 135.8, 134.6, 133.1 (d, *J*_C-F_ = 14.5 Hz), 132.0, 128.1, 127.2, 127.0, 126.7, 125.8, 122.8, 122.7, 122.25, 121.5 (d, *J*_C-F_ = 1.8 Hz), 121.0 (d, *J*_C-F_ = 2.9 Hz), 120.8, 120.7, 120.4, 120.4, 120.1, 113.2, 113.0, 110.9, 109.8, 109.1, 101.8, 101.7, 100.0, 99.7, 75.9, 70.5, 35.8 (d, *J*_C-F_ = 22.4 Hz), 31.4 (6C). ^19^F-NMR: 282 MHz, CD_2_Cl_2_; *δ*/ppm = −105.40. HRMS: (ESI+, *m*/*z*) calc. for C_49_H_46_FIrN_5_O_2_ [M + H]^+^: 948.3265, found: 948.3263. CD (mdeg, 0.05 M in MeOH), λ in nm: 418 (−5), 322 (20), 300 (−6), 285 (−2), 260 (−18), 251 (−13), 234 (−40).

Analytical data for Δ-(*S*)-**3a**: TLC: R*_f_* = 0.68 (*n*-pentane/EtOAc 2:1). ^1^H-NMR: 300 MHz, CD_2_Cl_2_; *δ*/ppm = 8.58 (s, 1H, H_arom._), 8.47 (s, 1H, H_arom._), 8.23 (s, 1H, H_arom._), 7.80 (s, 1H, H_arom._), 7.77 (d, ^3^*J* = 9.0 Hz, 1H, H_arom._), 7.62 (d, ^3^*J* = 8.9 Hz, 1H, H_arom._), 7.40 (dd, ^3^*J* = 9.1 Hz, ^4^*J* = 1.5 Hz, 1H, H_arom._), 7.36 (d, ^3^*J* = 8.0 Hz, 1H, H_arom._), 7.29 (dd, ^3^*J* = 8.9 Hz, ^4^*J* = 1.4 Hz, 1H, H_arom._), 7.17 (d, ^3^*J* = 8.1 Hz, 1H, H_arom._), 6.93–−6.75 (m, 7H, H_arom._), 6.61–6.53 (m, 2H, H_arom._), 6.25 (d, ^3^*J* = 8.7 Hz, 1H, H_arom._), 6.07 (dt, ^3^*J* = 7.5 Hz, ^4^*J* = 0.9 Hz, 1H, H_arom._), 5.98–5.90 (m, 2H, H_arom._), 5.59 (dd, ^3^*J* = 7.7 Hz, ^4^*J* = 0.8 Hz, 1H, H_arom._), 4.27 (dd, ^3^*J* = 10.5 Hz, ^3^*J* = 9.8 Hz, 1H, H_aliph._), 4.05 (t, ^3^*J* = 9.3 Hz, 1H, H_aliph._), 3.79 (t, ^3^*J* = 10.0 Hz, 1H, H_aliph._), 1.39 (s, 9H, H*_t_*_-butyl_), 1.23 (s, 9H, H*_t_*_-butyl_). ^13^C-NMR: 76 MHz, CD_2_Cl_2_; *δ*/ppm = 174.4 (d, *J*_C-F_ = 3.6 Hz), 174.3, 164.1 (d, *J*_C-F_ = 1.5 Hz), 161.5, 153.6, 152.7, 148.9 (d, *J*_C-F_ = 19.4 Hz), 144.3, 144.0, 140.1, 137.0, 136.8, 134.8, 133.1, 132.9, 132.4, 128.5, 127.8, 127.4, 127.0, 126.6, 123.4, 122.9, 122.3, 121.2, 121.1, 120.9, 120.5, 120.5, 120.2, 120.0 (d, *J*_C-F_ = 2.6 Hz), 119.9, 112.9, 112.6, 110.5, 103.7 (d, *J*_C-F_ = 8.2 Hz), 99.8, 99.5, 75.9, 71.1, 36.0, 35.8, 31.4 (d, *J*_C-F_ = 0.8 Hz), 31.2 (6C). ^19^F-NMR: 282 MHz, CD_2_Cl_2_; *δ*/ppm = ‒106.95. HRMS: (ESI+, *m*/*z*) calc. for C_49_H_46_FIrN_5_O_2_ [M + H]^+^: 948.3265, found: 948.3263. CD (mdeg, 0.05 M in MeOH), λ in nm: 417 (12), 324 (−25), 303 (23), 285 (10), 257 (21), 250 (20), 234 (73).

**Λ-(*S*)-3b andΔ-(*S*)-3b.***rac*-**IrBim** (46 mg, 48.6 µmol, 1.00 equiv.), (*S*)-**2** (13.7 mg, 53.5 µmol, 1.10 equiv.) and K_2_CO_3_ (20.7 mg, 0.15 mmol, 3.00 equiv.) were added to a Schlenk tube, the tube was evacuated for 5 min and absolute ethanol (2 mL, 0.025 m) was added. The tube was sealed, heated to 70 °C for 6 h under a nitrogen atmosphere and then diluted with CH_2_Cl_2_ (5 mL) and finally filtered through a short plug of Celite. The filtrate was concentrated under reduced pressure and the crude product was purified by silica gel column chromatography (*n*-pentane/EtOAc 6:1 → 1:1) to yield Λ-(*S*)-**3b** (23.9 mg, 24.5 μmol, 50%) and Δ-(*S*)-**3b** (24.0 mg, 24.6 μmol, 50%), both as brown solids.

Analytical data for Λ-(*S*)-**3b**: TLC: R*_f_* = 0.39 (*n*-pentane/EtOAc 2:1). ^1^H-NMR: 300 MHz, CD_2_Cl_2_; *δ*/ppm = 8.15 (d, ^4^*J* = 1.4 Hz, 1H, H_arom._), 7.71 (d, ^3^*J* = 7.8 Hz, 1H, H_arom._), 7.61 (d, ^4^*J* = 1.2 Hz, 1H, H_arom._), 7.49 (dd, ^3^*J* = 8.7 Hz, ^4^*J* = 1.7 Hz, 1H, H_arom._), 7.42 (d, ^3^*J* = 8.6 Hz, 1H, H_arom._), 7.41 (d, ^3^*J* = 8.5 Hz, 1H, H_arom._), 7.34 (d, ^3^*J* = 7.9 Hz, 1H, H_arom._), 7.14 (d, ^3^*J* = 8.6 Hz, 1H, H_arom._), 7.03–6.79 (m, 5H, H_arom._), 6.63–6.45 (m, 5H, H_arom._), 6.14 (d, ^4^*J* = 6.9 Hz, 2H, H_arom._), 6.03 (dd, ^3^*J* = 7.7 Hz, ^4^*J* = 0.6 Hz, 1H, H_arom._), 5.92 (ddd, ^3^*J* = 13.0 Hz, ^3^*J* = 7.9 Hz, ^4^*J* = 1.0 Hz, 1H, H_arom._), 4.97 (dd, ^3^*J* = 9.5 Hz, ^4^*J* = 4.3 Hz, 1H, H_aliph._), 4.82 (t, ^3^*J* = 9.2 Hz, 1H, H_aliph._), 4.18 (s, 3H, H_aliph._), 3.96 (dd, ^3^*J* = 8.6 Hz, ^4^*J* = 4.2 Hz, 1H, H_aliph._), 3.78 (s, 3H, H_aliph._), 1.44 (s, 9H, H*_t_*_-butyl_), 1.28 (s, 9H, H*_t_*_-butyl_). ^13^C-NMR: 75 MHz, CD_2_Cl_2_; *δ*/ppm = 173.7, 165.4, 165.0, 163.2, 162.4, 155.8, 150.0, 147.7, 147.6, 141.8, 141.3, 140.9, 137.2, 136.7, 135.7, 134.2, 133.9, 133.4, 132.7, 132.5, 129.1 (d, *J*_C-F_ = 3.8 Hz), 127.4, 126.8, 124.9, 124.9, 121.3, 121.3, 121.1, 121.0, 120.2, 114.4, 112.4, 109.8, 109.0, 101.7 (d, *J*_C-F_ = 6.7 Hz), 99.5, 99.2, 75.6, 75.6, 70.8, 35.5, 35.3, 32.6, 32.2 (3C), 32.2 (3C), 31.8, 30.3. ^19^F-NMR: 282 MHz, CD_2_Cl_2_; *δ*/ppm = −105.37. HRMS: (ESI+, *m*/*z*) calc. for C_51_H_50_FIrN_5_O_2_ [M + H]^+^: 976.3578, found: 976.3576. CD (mdeg, 0.05 M in MeOH), λ in nm: 441 (−12), 354 (65), 331 (54), 319 (83), 296 (−46), 292 (−44), 270 (−3), 267 (−5), 252 (29), 224 (−73).

Analytical data for Δ-(*S*)-**3b**: TLC: R*_f_* = 0.28 (*n*-pentane/EtOAc 2:1). ^1^H-NMR: 300 MHz, CD_2_Cl_2_; *δ*/ppm = 8.25 (dd, ^4^*J* = 1.4 Hz, ^4^*J* = 0.8 Hz, 1H, H_arom._), 7.85 (d, ^4^*J* = 1.4 Hz, 1H, H_arom._), 7.66 (dd, ^3^*J* = 7.9 Hz, ^4^*J* = 0.8 Hz, 1H, H_arom._), 7.52–7.46 (m, 3H, H_arom._), 7.43 (dd, ^3^*J* = 8.7 Hz, ^4^*J* = 1.7 Hz, 1H, H_arom._), 7.34 (d, ^3^*J* = 8.7 Hz, 1H, H_arom._), 6.93–6.74 (m, 7H, H_arom._), 6.60 (dt, ^3^*J* = 7.6 Hz, ^4^*J* = 1.3 Hz, 1H, H_arom._), 6.53 (dt, ^3^*J* = 7.6 Hz, ^4^*J* = 1.2 Hz, 1H, H_arom._), 6.28 (d, ^3^*J* = 8.6 Hz, 1H, H_arom._), 6.21 (dd, ^3^*J* = 7.6 Hz, ^4^*J* = 0.8 Hz, 1H, H_arom._), 6.07 (dt, ^3^*J* = 7.5 Hz, ^4^*J* = 1.2 Hz, 1H, H_arom._), 5.92–5.85 (m, 2H, H_arom._), 4.30 (s, 3H, H_aliph._), 4.21–4.12 (m, 1H, H_aliph._), 4.16 (s, 3H, H_aliph._), 4.07–3.99 (m, 2H, H_aliph._), 1.38 (s, 9H, H*_t_*_-butyl_), 1.23 (s, 9H, H*_t_*_-butyl_). 13C-NMR: 76 MHz, CD_2_Cl_2_; *δ*/ppm = 182.0, 179.0, 174.6 (d, *J*_C-F_ = 3.8 Hz), 165.0, 164.8, 163.1, 163.1, 161.5, 154.1, 151.0, 147.6 (d, *J*_C-F_ = 86.0 Hz), 141.3 (d, *J*_C-F_ = 25.3 Hz), 140.7, 136.5, 136.4, 135.3, 134.1, 133.9 (d, *J*_C-F_ = 20.0 Hz), 132.4 (d, *J*_C-F_ = 13.6 Hz), 129.0 (d, *J*_C-F_ = 28.1 Hz), 128.4, 127.6, 127.5, 124.7, 121.5, 121.4, 121.0, 120.2, 120.2, 119.8, 114.1, 113.7, 109.6, 109.5, 103.8, 103.7, 99.2, 98.9, 75.6, 69.9, 35.4, 35.3, 32.4, 32.4, 32.1 (d, *J*_C-F_ = 0.8 Hz, 3C), 31.9 (3C), 31.2. ^19^F-NMR: 282 MHz, CD_2_Cl_2_; *δ*/ppm = −107.00. HRMS: (ESI+, *m*/*z*) calc. for C_51_H_50_FIrN_5_O_2_ [M + H]^+^: 976.3578, found: 976.3576. CD (mdeg, 0.05 M in MeOH), λ in nm: 444 (23), 321 (−32), 295 (53), 274 (25), 272 (26), 255 (−10), 252 (−11), 224 (78).

**Λ- andΔ-IrInd.** Λ-(*S*)-**3a** (97 mg, 0.10 mmol, 1.00 equiv.) or Δ-(*S*)-**3a** (100 mg, 0.11 mmol, 1.00 equiv) was dissolved in MeCN (5 mL, 0.02 m) and TFA (for Λ-(*S*)-**3a**: 46 µL, 0.60 mmol; for Δ-(*S*)-**3a**: 51 µL, 0.66 mmol; 6.00 equiv.) was added dropwise. The solution was stirred for 30 min under a nitrogen atmosphere at room temperature and then concentrated under reduced pressure. The residue was redissolved in MeCN (5 mL, 0.02 m), and NH_4_PF_6_ (for Λ-(*S*)-**3a**: 326 mg, 2.0 mmol; for Δ-(*S*)-**3a**: 359 mg, 2.20 mmol; 20.0 equiv.) was added in one portion. The suspension was stirred at room temperature for 30 min, concentrated and purified via silica gel column chromatography (CH_2_Cl_2_/MeCN 20:1 → 10:1) to yield Λ-**IrInd** (80.7 mg, 88.0 µmol, 86%) or Δ-**IrInd** (94.6 mg, 103 µmol, 98%) as a yellow, crystalline solid.

Analytical data for Λ-**IrInd**: HPLC: er > 99% (Daicel Chiralpak IB N-5 column, 250 × 4.6 m^2^, 254 nm, 25 °C, 0.6 mL/min, H_2_O (+0.1% TFA)/MeCN 60:40 to 50:50 in 180 min, holding 50% MeCN for 250 min, t_r_ (major) = 186.1 min, t_r_ (minor) = 182.1 min). CD (mdeg, 0.05 M in MeOH), λ in nm: 403 (−24), 362 (31), 351 (23), 317 (97), 296 (−54), 293 (−53), 263 (−150), 245 (54), 240 (42), 217 (263). All other analytical data were in agreement with the racemic complex.

Analytical data for **Δ-IrInd**: HPLC: er > 99% (Daicel Chiralpak IB N-5 column, 250 × 4.6 m^2^, 254 nm, 25 °C, 0.6 mL/min, H_2_O (+0.1% TFA)/MeCN 60:40 to 50:50 in 180 min, holding 50% MeCN for 250 min, t_r_ (major) = 182.1 min, t_r_ (minor) = 186.1 min). CD (mdeg, 0.05 M in MeOH), λ in nm: 402 (20), 365 (−11), 352 (−5), 317 (−41), 263 (102), 245 (−12), 240 (−3), 213 (−175). All other analytical data were in agreement with the racemic complex.

**Λ- andΔ-IrBim.** Λ-(*S*)-**3b** (24.0 mg, 24.6 µmol, 1.00 equiv.) or Δ-(*S*)-**3b** (80.0 mg, 82.0 µmol, 1.00 equiv) was dissolved in MeCN (for Λ-(*S*)-**3b**: 1.25 mL; for Δ-(*S*)-**3b**: 4.1 mL, 0.02 m) and TFA (for Λ-(*S*)-**3b**: 11.5 µL, 0.15 mmol; for Δ-(*S*)-**3b**: 38 µL, 0.49 mmol; 6.00 equiv.) was added dropwise. The solution was stirred for 30 min under a nitrogen atmosphere at room temperature and then concentrated under reduced pressure. The residue was redissolved in MeCN (for Λ-(*S*)-**3b**: 1.25 mL; for Δ-(*S*)-**3b**: 4.1 mL, 0.02 m) and NH_4_PF_6_ (for Λ-(*S*)-**3b**: 81.5 mg, 0.50 mmol; for Δ-(*S*)-**3b**: 267 mg, 1.64 mmol; 20.0 equiv.) was added in one portion. The suspension was stirred at room temperature for 30 min, concentrated and purified via silica gel column chromatography (CH_2_Cl_2_/MeCN 20:1 → 10:1) to yield Λ-**IrBim** (22.5 mg, 23.8 µmol, 97%) or Δ-**IrBim** (59.8 mg, 63.2 µmol, 77%) as yellow, crystalline solid.

Analytical data for Λ-**IrBim**: HPLC: er > 99% (Daicel Chiralpak IB N-5 column, 250 × 4.6 m^2^, 254 nm, 25 °C, 0.6 mL/min, H_2_O (+0.1% TFA)/MeCN 60:40 to 50:50 in 180 min, holding 50% MeCN for 250 min, t_r_ (major) = 190.2 min, t_r_ (minor) = 177.0 min). CD (mdeg, 0.05 M in MeOH), λ in nm: 427 (−7), 342 (38), 293 (−30), 279 (−20), 265 (−27), 247 (6), 239 (−5), 229 (9), 220 (−14), 212 (79). All other analytical data were in agreement with the racemic complex.

Analytica data for Δ-**IrBim**: HPLC: er = >99% (Daicel Chiralpak IB N-5 column, 250 × 4.6 mm, 254 nm, 25 °C, 0.6 mL/min, H_2_O (+0.1% TFA)/MeCN 60:40 to 50:50 in 180 min, holding 50% MeCN for 250 min, t_r_ (major) = 177.0 min, t_r_ (minor) = 190.2 min). CD (mdeg, 0.05 M in MeOH), λ in nm: 427 (9), 341 (−52), 292 (45), 278 (29), 267 (39), 248 (−7), 239 (9), 227 (−17), 221 (13), 212 (−109). All other analytical data were in agreement with the racemic complex.

**Asymmetric Friedel–Crafts reaction.** A Schlenk tube was charged with the iridium catalyst (6.0 µmol, 2 mol%), followed by THF (0.3 mL, 1.0 M) under a nitrogen atmosphere. The α,β-unsaturated 2-acyl imidazole **4** (45.1 mg, 0.30 mmol, 1.00 eq.) was added to the suspension in one portion, whereupon a black solution formed. The solution was stirred for 20 min at room temperature and subsequently treated with indole (87.8 mg, 0.75 mmol, 2.50 eq.). The resulting solution was stirred at room temperature until complete consumption of α,β-unsaturated 2-acyl imidazole **4** was detected by TLC. The solution was concentrated under reduced pressure and purified via silica gel column chromatography (*n*-pentane/EtOAc 1.5:1 to 1:1) to yield the Friedel–Crafts adduct (*S*)- or (*R*)-**5** as a white solid. The enantiomeric excess was determined by chiral HPLC analysis (Daicel Chiralpak IC column, 250 × 4.6 m^2^, absorbance at 254 nm, column temperature 40 °C, mobile phase *n*-hexane/*i*-PrOH 90:10, flow rate 0.5 mL/min, t_r_ (major) = 23.7 min, t_r_ (minor) = 28.3 min).

**Asymmetric Nazarov cyclization.** A Schlenk tube was charged with the iridium catalyst (1.8 µmol, 2 mol%), an *E*/*Z* mixture of indole-functionalized α-unsaturated β-ketoester **6** (27.5 mg, 0.09 mmol, 1.00 eq.) and HFIP (0.3 mL, 0.3 m) under a nitrogen atmosphere. The mixture was homogenized via sonication (1 min) and subsequently placed in a preheated oil bath at 50 °C. The solution was stirred at 50 °C until the complete consumption of used α-unsaturated β-ketoester **6** was indicated via TLC analysis. The solvent was then removed under reduced pressure and the diastereomeric ratio was determined via ^1^H-NMR analysis. The crude product was redissolved in CH_2_Cl_2_, transferred into a 10 mL-flask and basic Al_2_O_3_ (Sigma Aldrich, Darmstadt, Germany, 58 Å pore size, pH 9.5 ± 0.5 in water, 140 mg) was added in one portion. The suspension was stirred for 24 h and filtered over a cotton plug subsequently. The obtained solution was concentrated under reduced pressure and purified via silica gel column chromatography (*n*-pentane/EtOAc 3:1 to 2:1) to give the desired cyclized product **7**. The Al_2_O_3_-equilibrated diastereomeric ratio was determined via ^1^H-NMR analysis and the enantiomeric excess for the major diastereomer (*trans*-diastereomer **7**) was determined via chiral HPLC analysis (Daicel Chiralpak AD-H column, 250 × 4.6 m^2^, absorbance at 254 nm, column temperature 25 °C, mobile phase *n*-hexane/*i*-PrOH 90:10, flow rate 0.6 mL/min, t_r_ (major) = 21.0 min, t_r_ (minor) = 30.3 min).

**Single-crystal X-ray diffraction.** Crystal structure data can be accessed via the Cambridge Crystallographic Data Centre (CCDC) under the deposition numbers 2069740 (Λ-**IrInd**), 2069741 (*rac*-**IrBim**), and 2069742 (Λ-(*S*)-**3b**).

## 4. Conclusions

In conclusion, we introduced two new bis-cyclometalated iridium(III) catalysts containing two inert cyclometalated 6-*tert*-butyl-2-phenyl-2*H*-indazole bidentate ligands or two inert cyclometalated 5-*tert*-butyl-1-methyl-2-phenylbenzimidazoles, together with two labile acetonitriles and a hexafluorophosphate counterion. These complexes complement previously reported related benzoxazole and benzothiazole complexes and thereby expand the family of bis-cyclometalated chiral-at-iridium catalysts for application in asymmetric catalysis. The reasons for the differences in the catalytic activity and stereoselectivity of the catalysts after replacing benzoxazole or benzothiazole with indazole or benzimidazole moieties need to be examined. Future work will also investigate their properties as catalysts for asymmetric photochemistry.

## Figures and Tables

**Table 1:** Catalytic enantioselective conjugate addition of indole to 2-acyl imidazole **4** *^a^*.

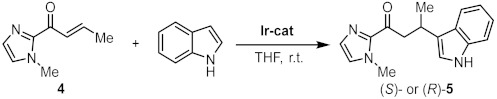					
Entry	Catalyst	Loading (mol%)	Time (h)	Yield*^b^*	ee *^c^*
1	Λ-**IrInd**	2.0	29	84 (*S*)	98.5
2	Δ-**IrBim**	2.0	49	81 (*R*)	98
3 *^d^*	Λ-**IrO**	1.0	20	97 (*S*)	96
4 *^e^*	Λ-**IrS**	1.0	40	94 (*S*)	99

*^a^* Reaction conditions: 2-Acyl imidazole **4** (0.30 mmol), indole (0.75 mmol), and iridium catalyst (1.0 or 2.0 mol%) in THF (0.3 mL) stirred under nitrogen at room temperature for the indicated time. *^b^* Isolated yields of (*S*)- or (*R*)-**5**. *^c^* Determined by HPLC on a chiral stationary phase. *^d^* Taken from ref. [4]. *^e^* Taken from ref. [44].

**Table 2:** Catalytic asymmetric Nazarov cyclization *^a^*.

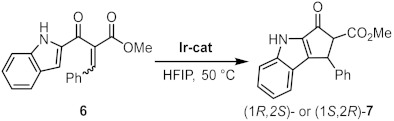					
Entry	Catalyst (mol%)	Time (h)	Yield*^b^*	*trans*/*cis*	ee *^c^*
1 *^d^*	Λ-**IrS** (2.0)	7	75% (1*R*,2*S*)	15:1	93
2	Λ-**IrInd** (2.0)	24	46% (1*R*,2*S*)	11:1	96
3	Δ-**IrBim** (2.0)	24	73% (1*S*,2*R*)	12.5:1	94

*^a^* Reaction conditions: Ketoester 6 (0.09 mmol) with iridium catalyst (2.0 mol%) in hexafluoroisopropanol (HFIP, 0.3 mL), first sonicated and then stirred under a nitrogen atmosphere for the indicated time. Afterwards, the solvent was exchanged for CH2Cl2 and the mixture was stirred in the presence of basic Al2O3 at room temperature for 24 h. *^b^* Isolated yields of (S)- or (R)-5. *^c^* Determined by HPLC on a chiral stationary phase. *^d^* Taken from ref. [51].

## Data Availability

All data are contained within the article and the Supplementary Materials.

## Supplementary Materials

The following are available online, NMR spectra, HPLC traces, and crystallographic data.

## Sample Availability

Samples of the compounds are not available from the authors.
