# Supplementary material for: Bis-Cyclometalated Indazole and Benzimidazole Chiral-at-Iridium Complexes: Synthesis and Asymmetric Catalysis
Source: Molecules. 2021 Mar 24;26(7):1822. doi: 10.3390/molecules26071822 (PMC8037582; doi:10.3390/molecules26071822)
Supplement: Supplementary file 1 [file molecules-26-01822-s001.pdf]

Supporting Information

Bis-Cyclometallated Indazole and Benzimidazole Chiral-at-Iridium Complexes: Synthesis and Asymmetric Catalysis

Sebastian Brunen, Yvonne Grell, Philipp S. Steinlandt, Klaus Harms, and Eric Meggers*

Fachbereich Chemie, Philipps-Universität Marburg, Hans-Meerwein-Straße 4, 35043 Marburg, Germany

*Email: meggers@chemie.uni-marburg.de

Contents

1. **NMR Spectra** S2
2. **HPLC Traces** S14
3. **Single Crystal X-Ray Diffraction** S17

1. NMR Spectra

rac-IrInd

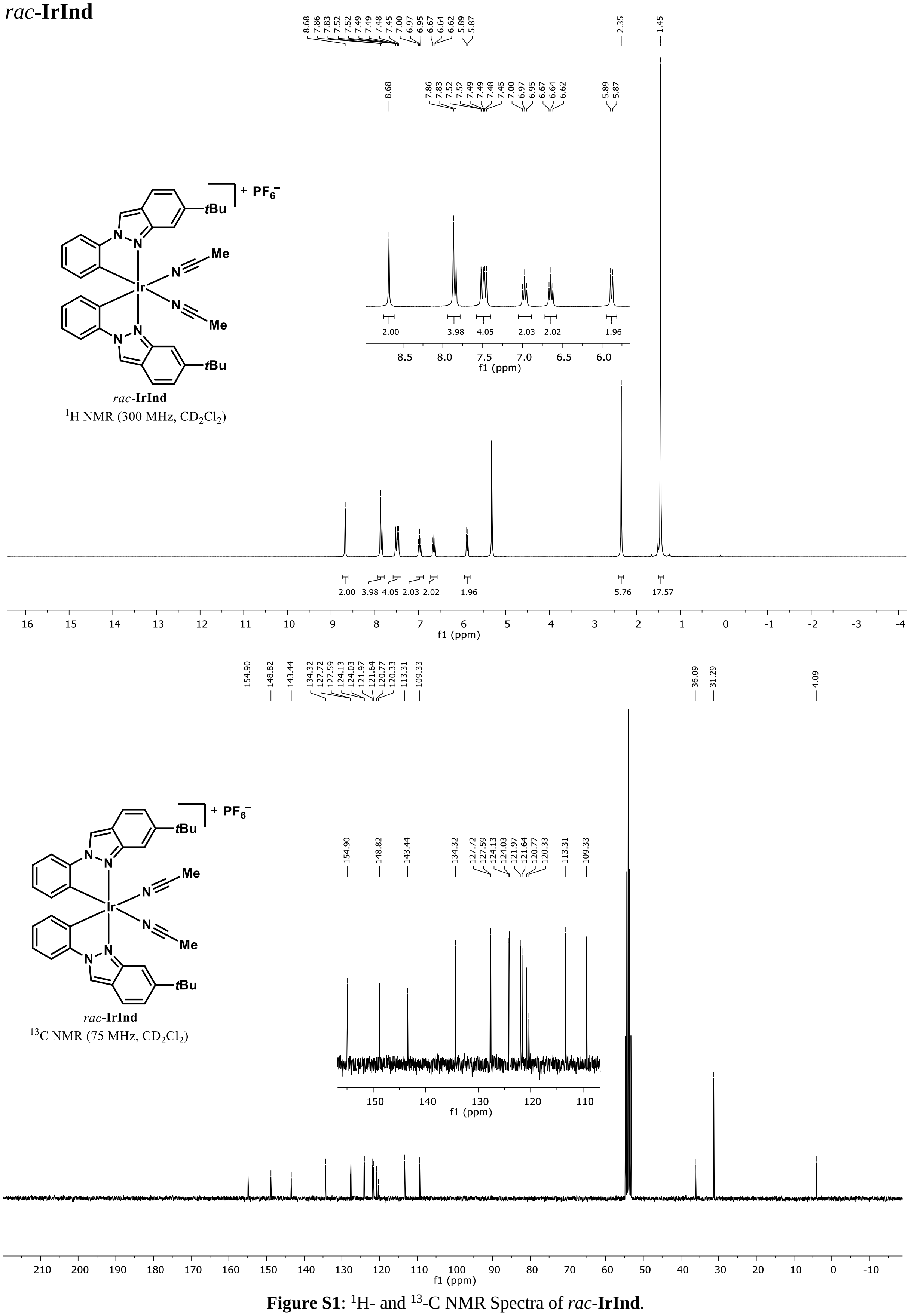

Figure S1: ^{1}H- and ^{13}-C NMR Spectra of *rac*-**IrInd**.

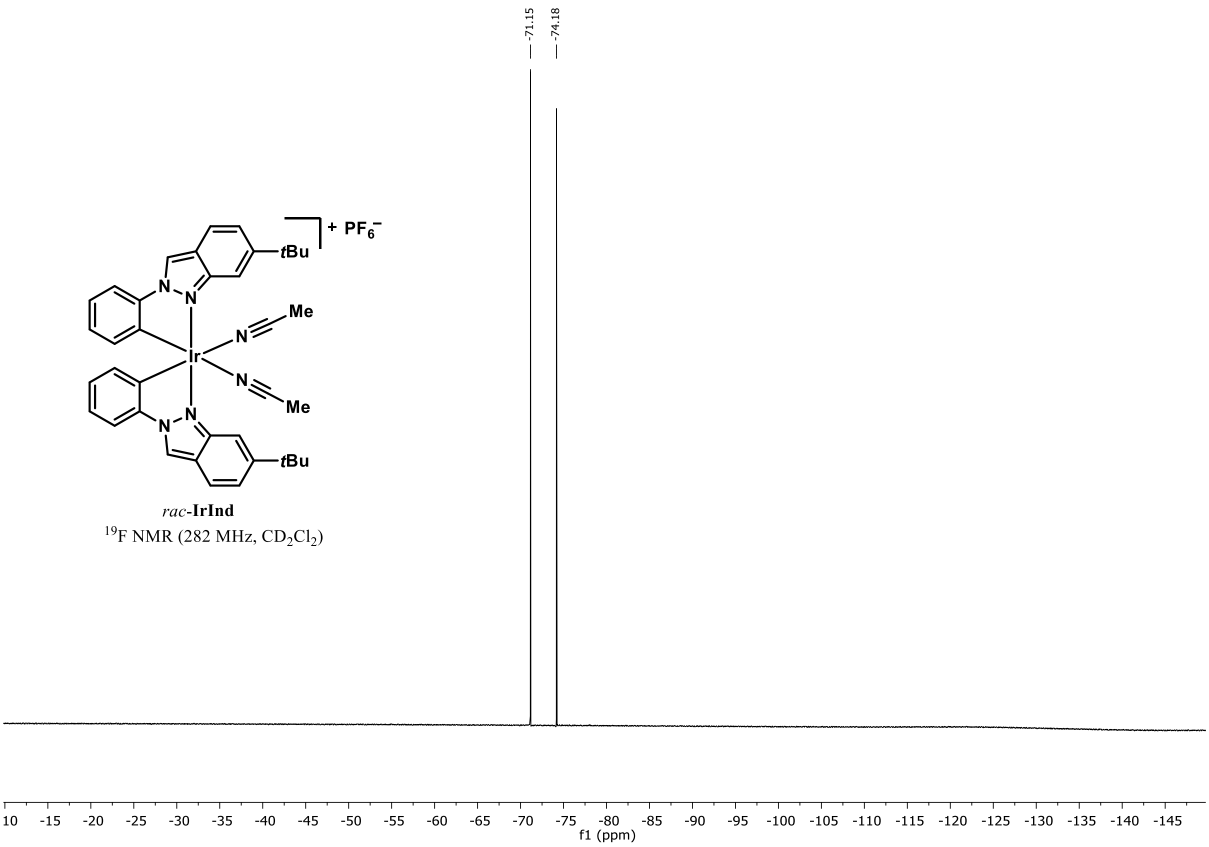

Figure S2: ^{19}F NMR Spectrum of *rac*-**IrInd**.

rac-**IrBim**

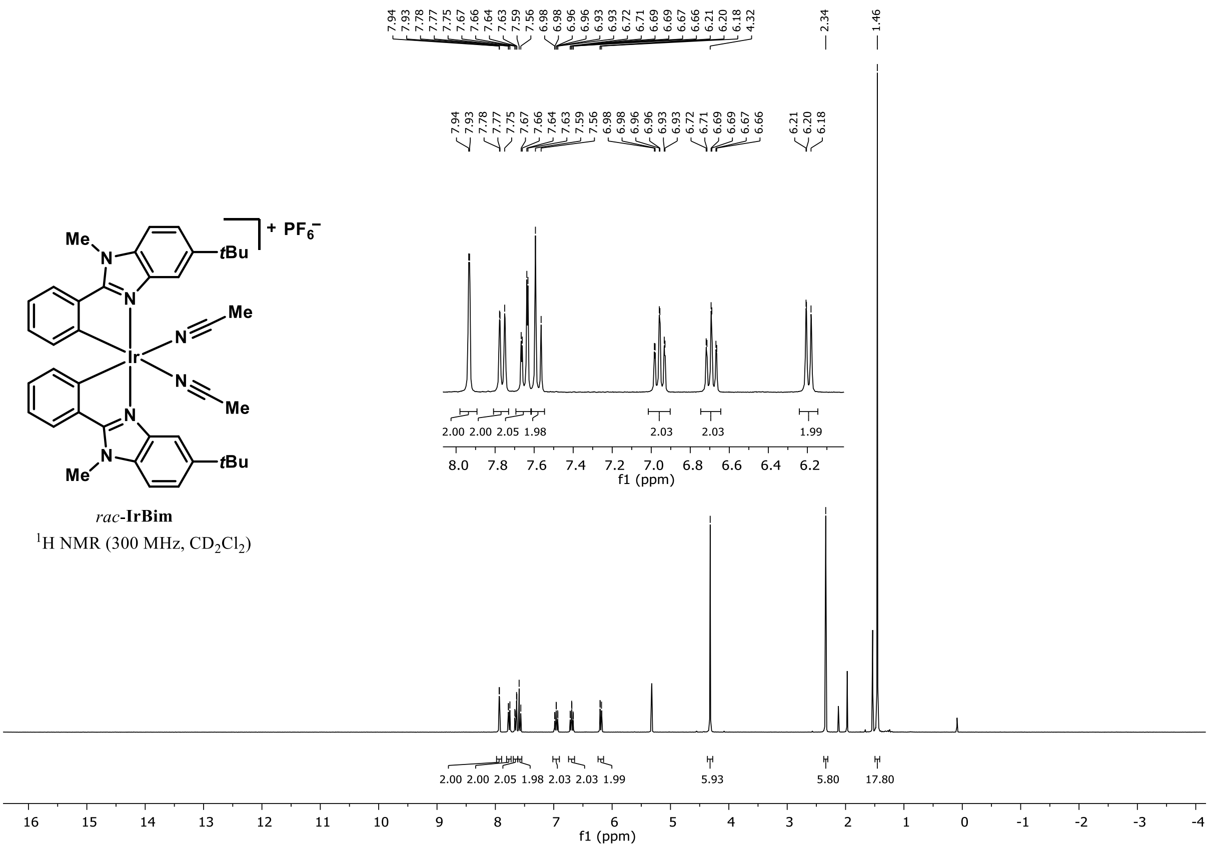

Figure S3: ^{1}H NMR Spectrum of *rac*-**IrBim**.

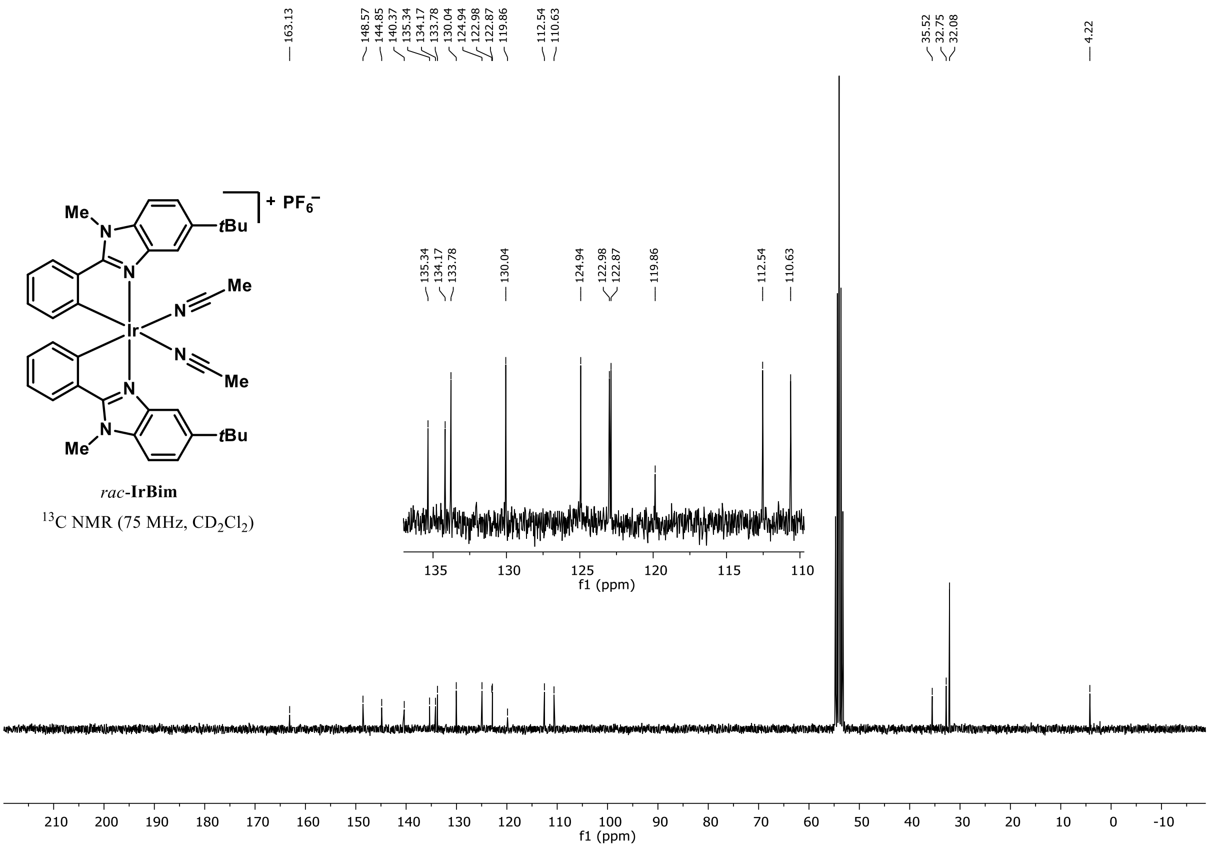

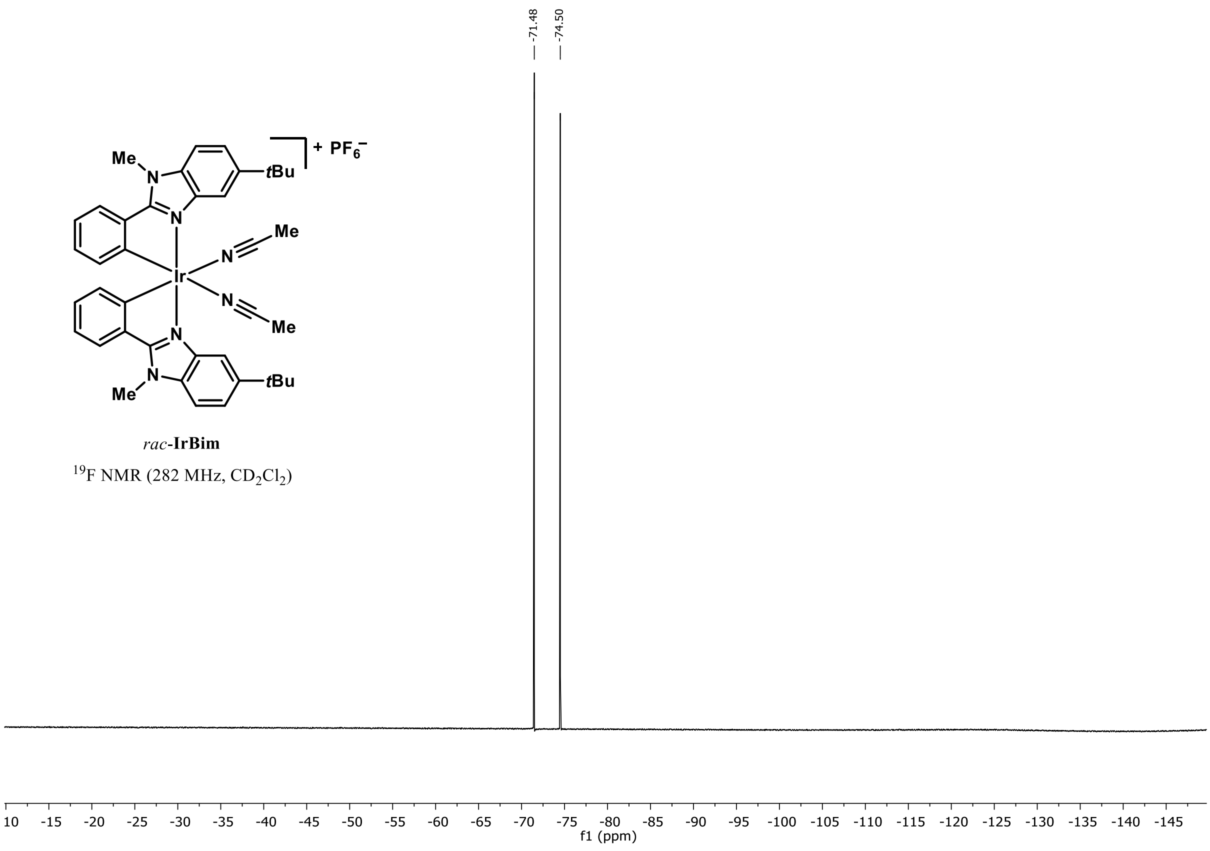

Figure S4: ^{13}C and ^{19}F NMR Spectra of *rac*-**IrBim**.

Λ-(*S*)-3a

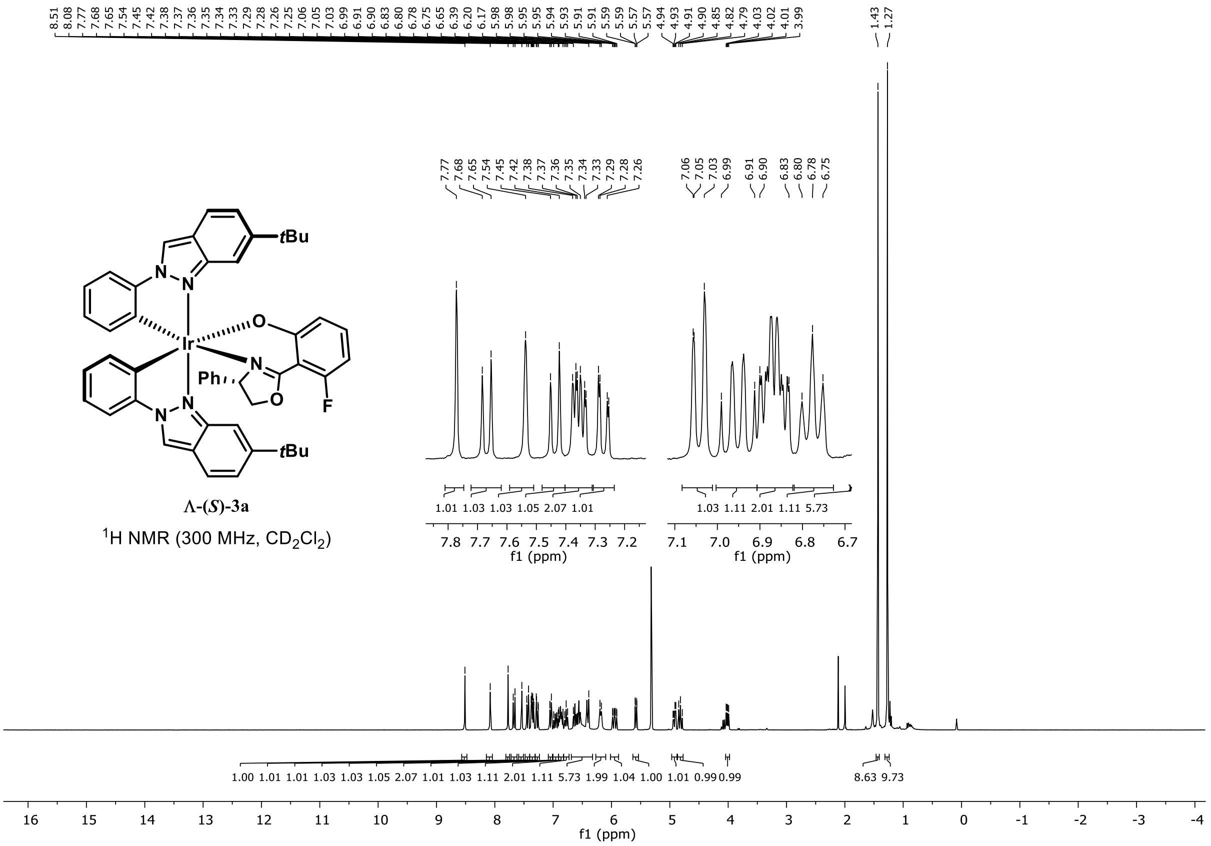

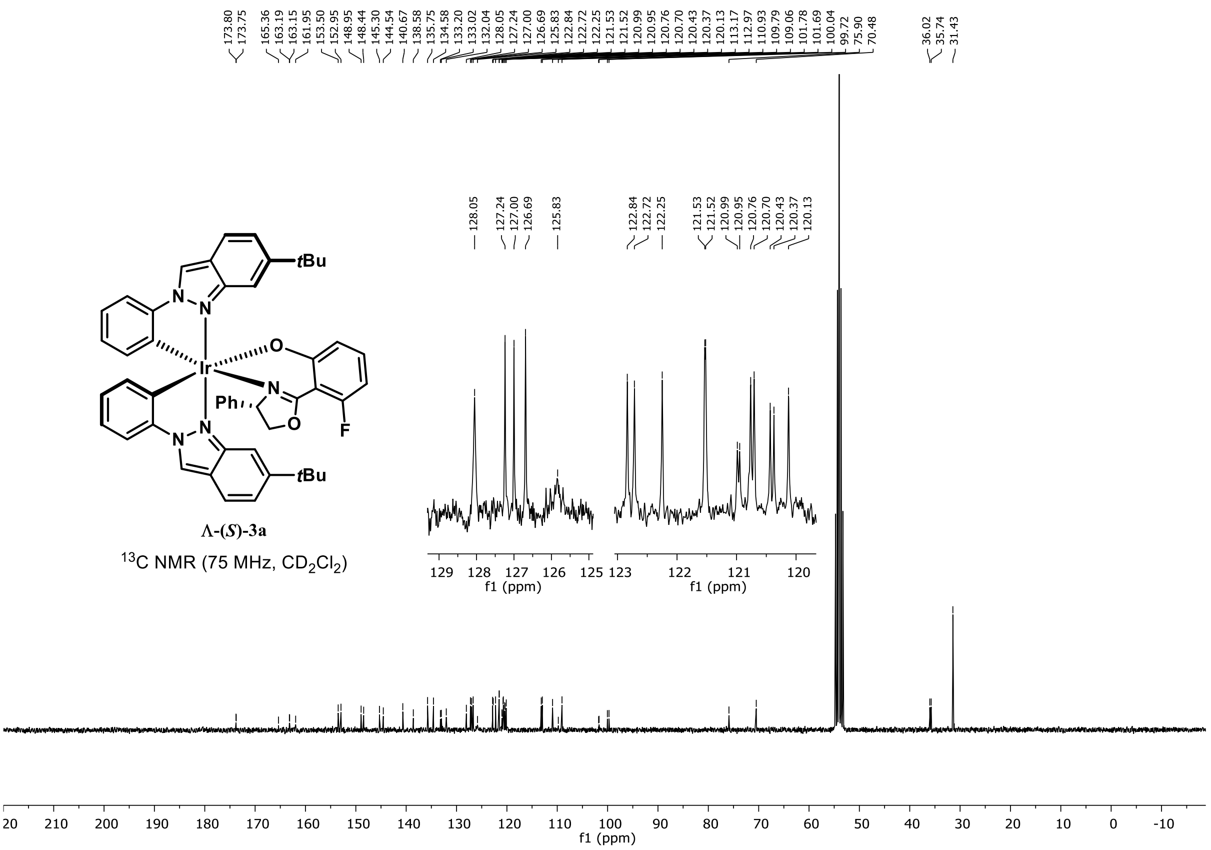

Figure S5: ^{1}H- and ^{13}-C NMR Spectra of Λ-(*S*)-**3a**.

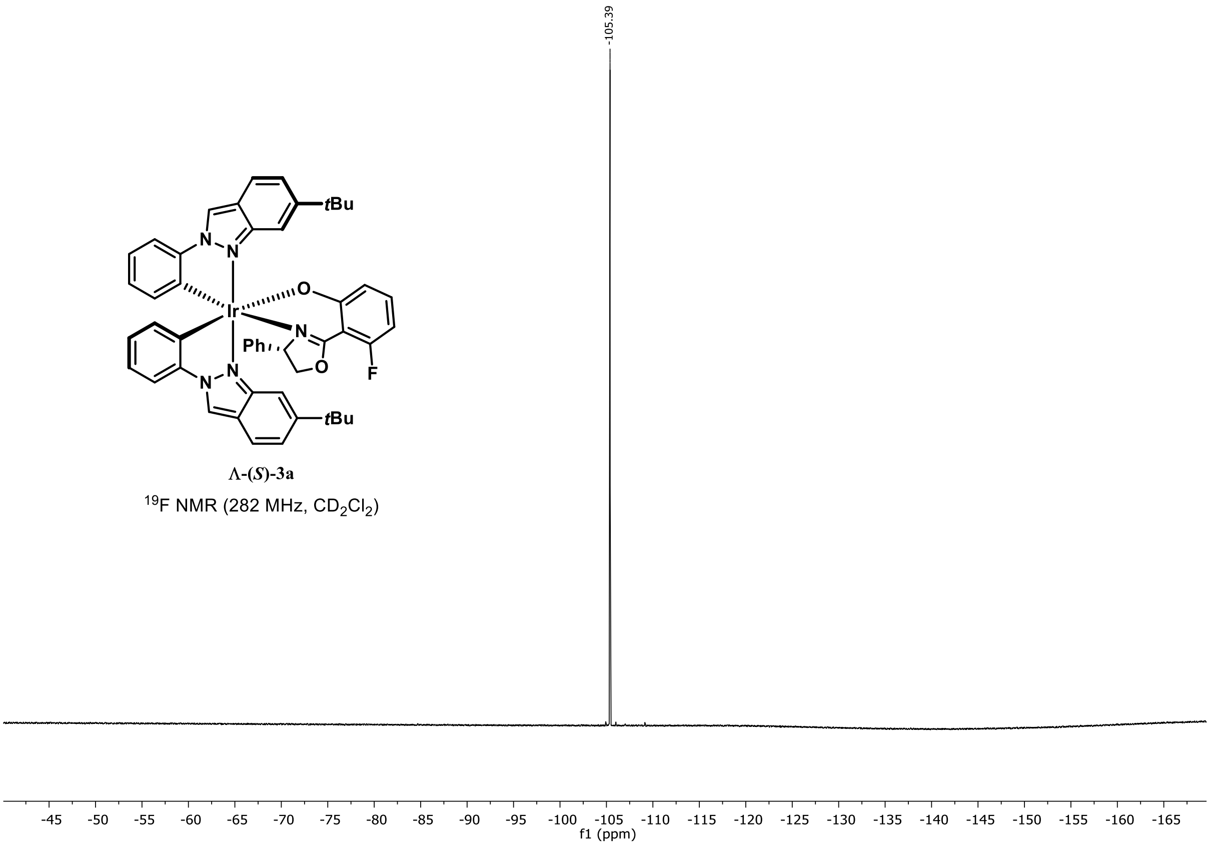

Figure S6: ^{19}F NMR Spectrum of Λ-(*S*)-**3a**.

Δ-(*S*)-3a

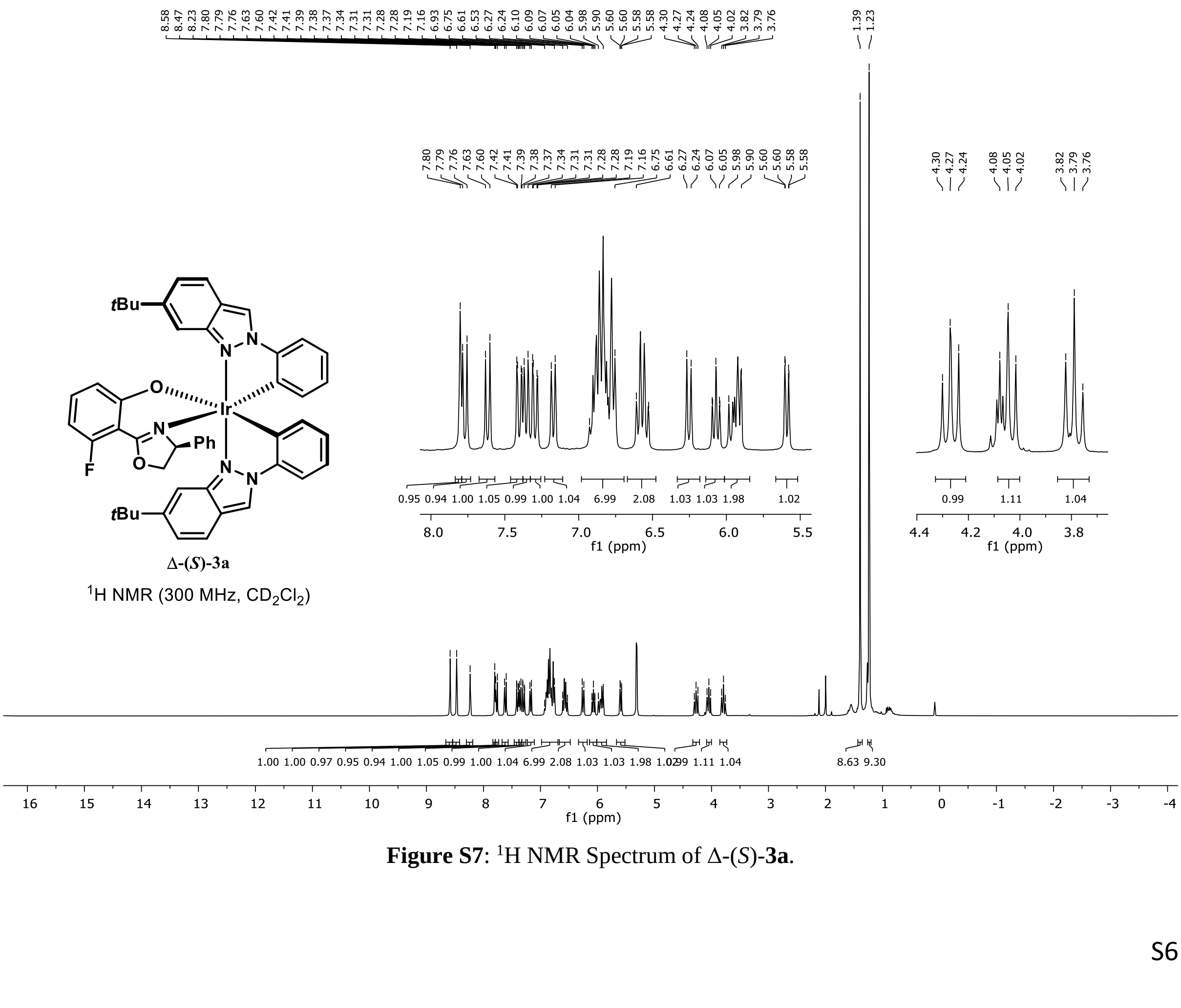

Figure S7: ^{1}H NMR Spectrum of Δ-(*S*)-**3a**.

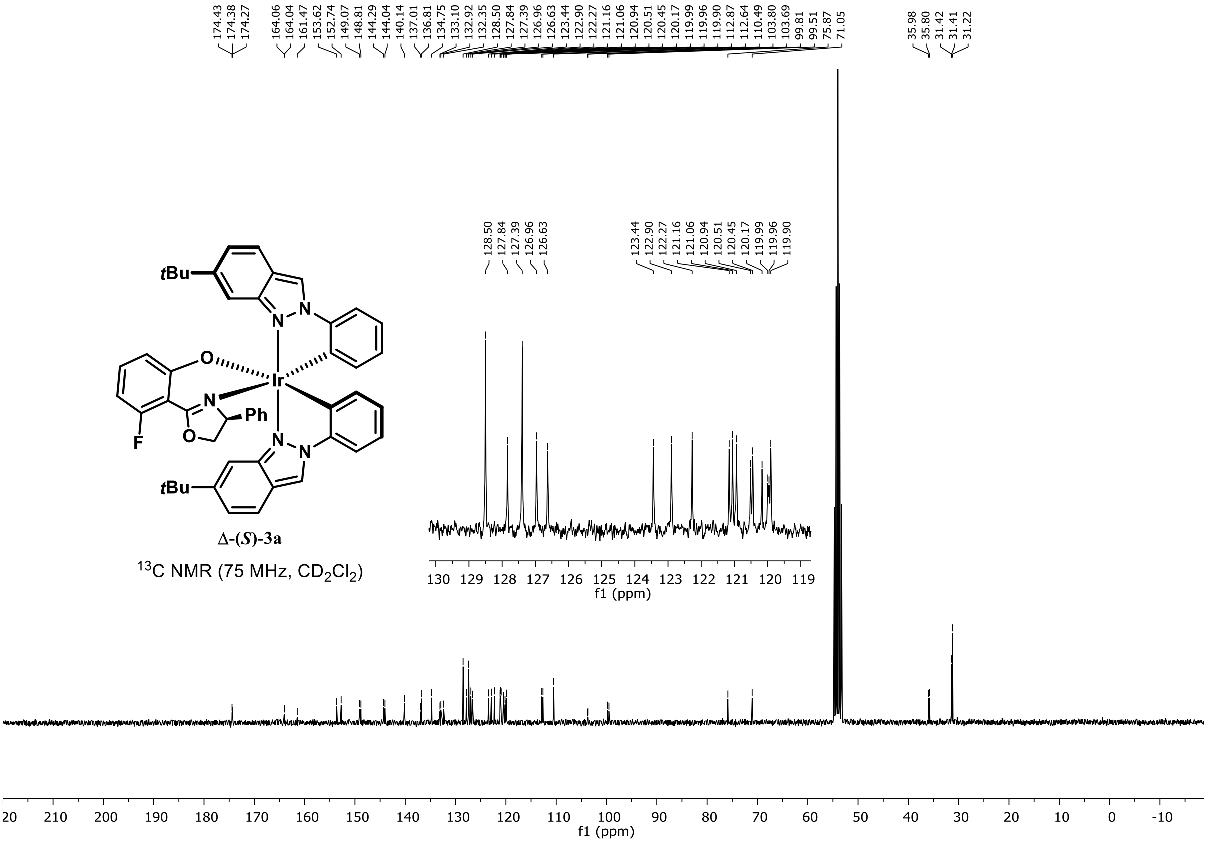

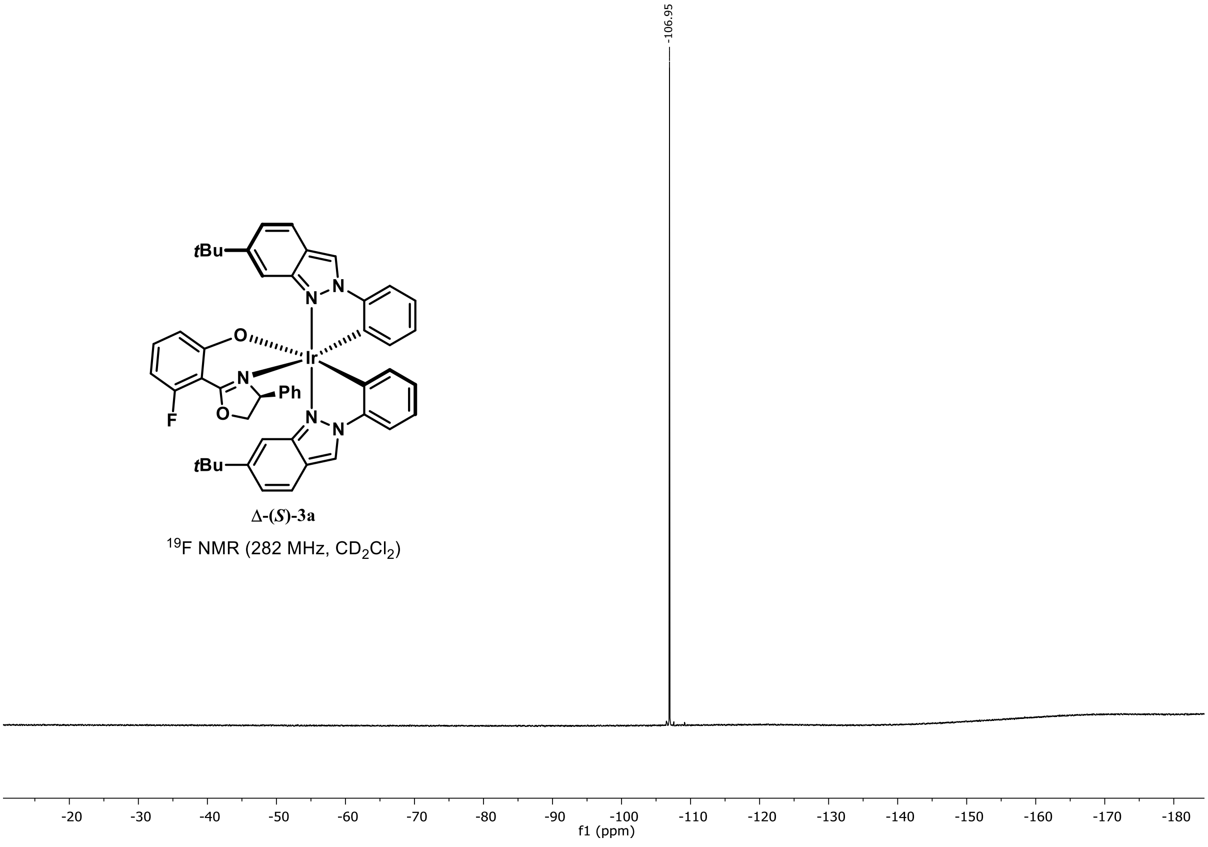

Figure S8: ^{13}C and ^{19}F NMR Spectra of Δ-(*S*)-**3a**.

Λ-(*S*)-**3b**

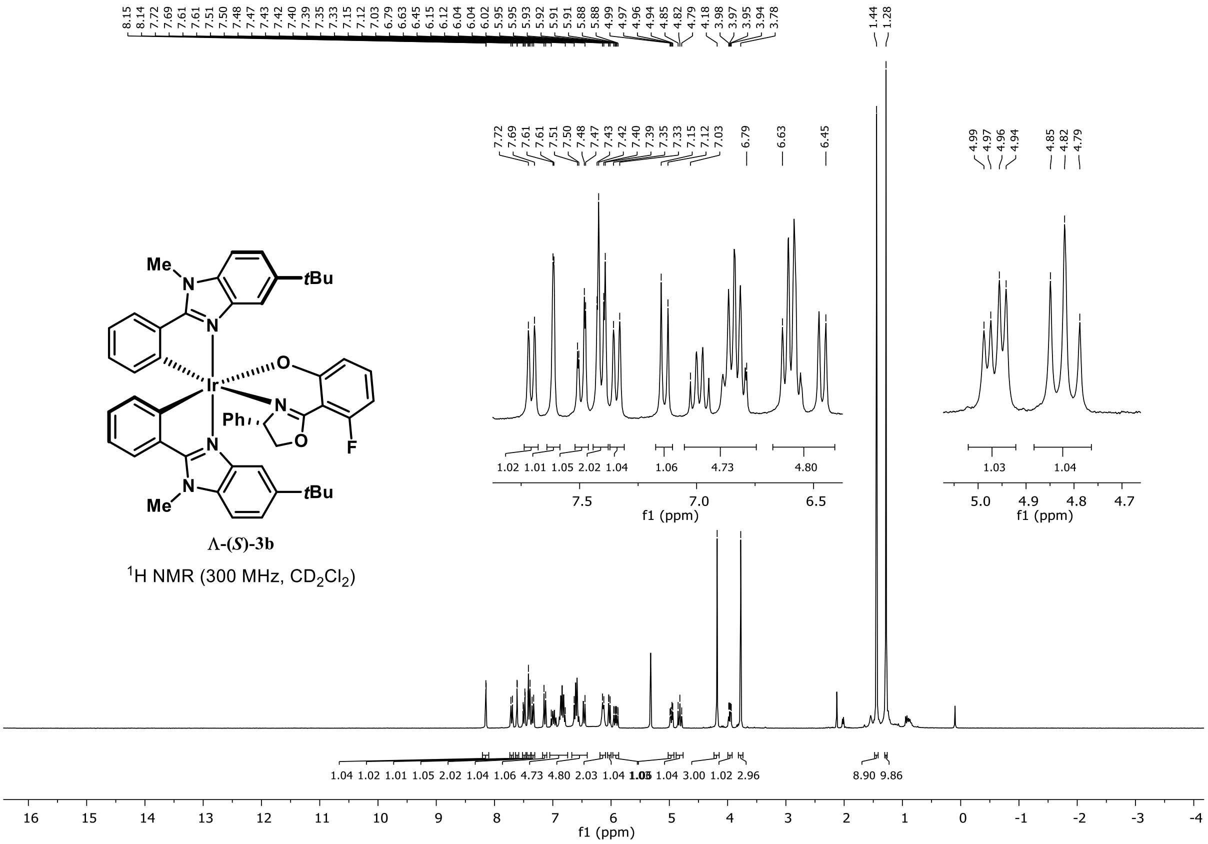

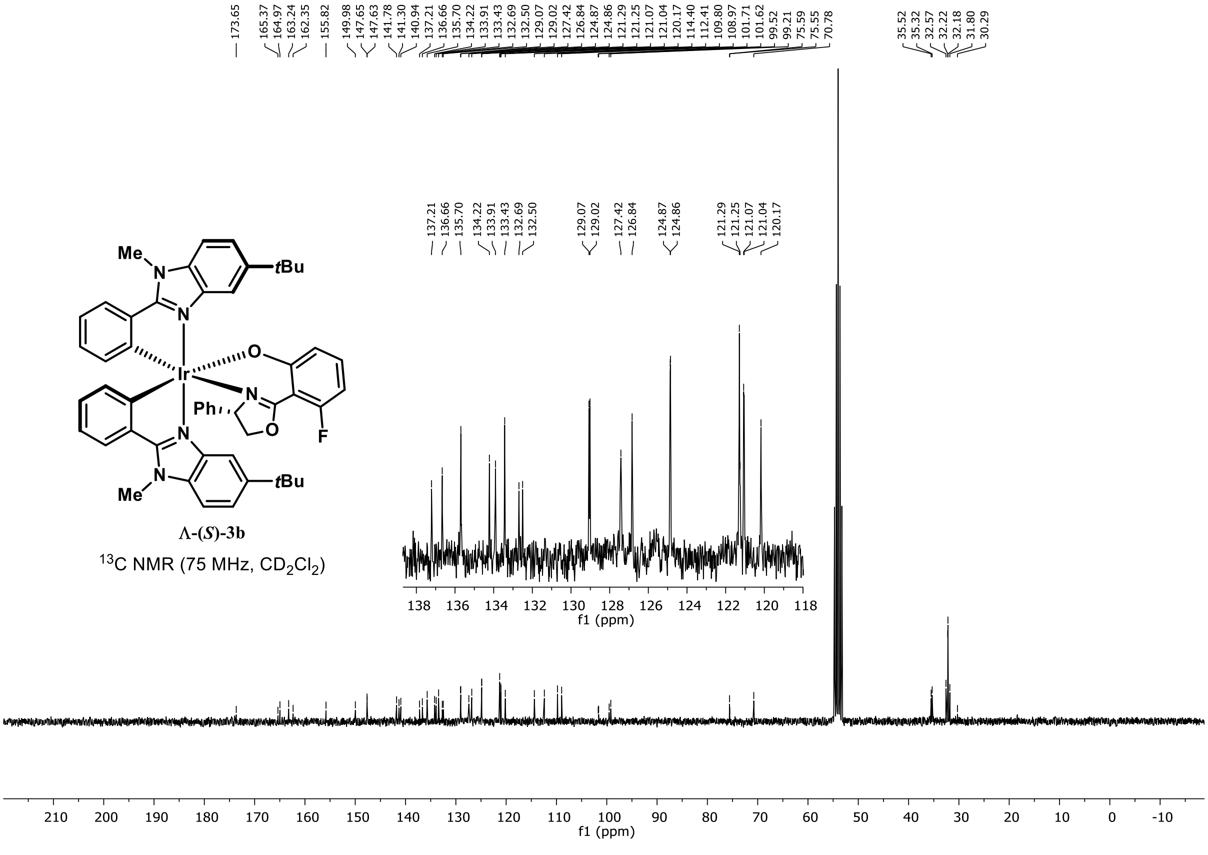

Figure S9: ^{1}H- and ^{13}-C NMR Spectra of Λ-(*S*)-**3b**.

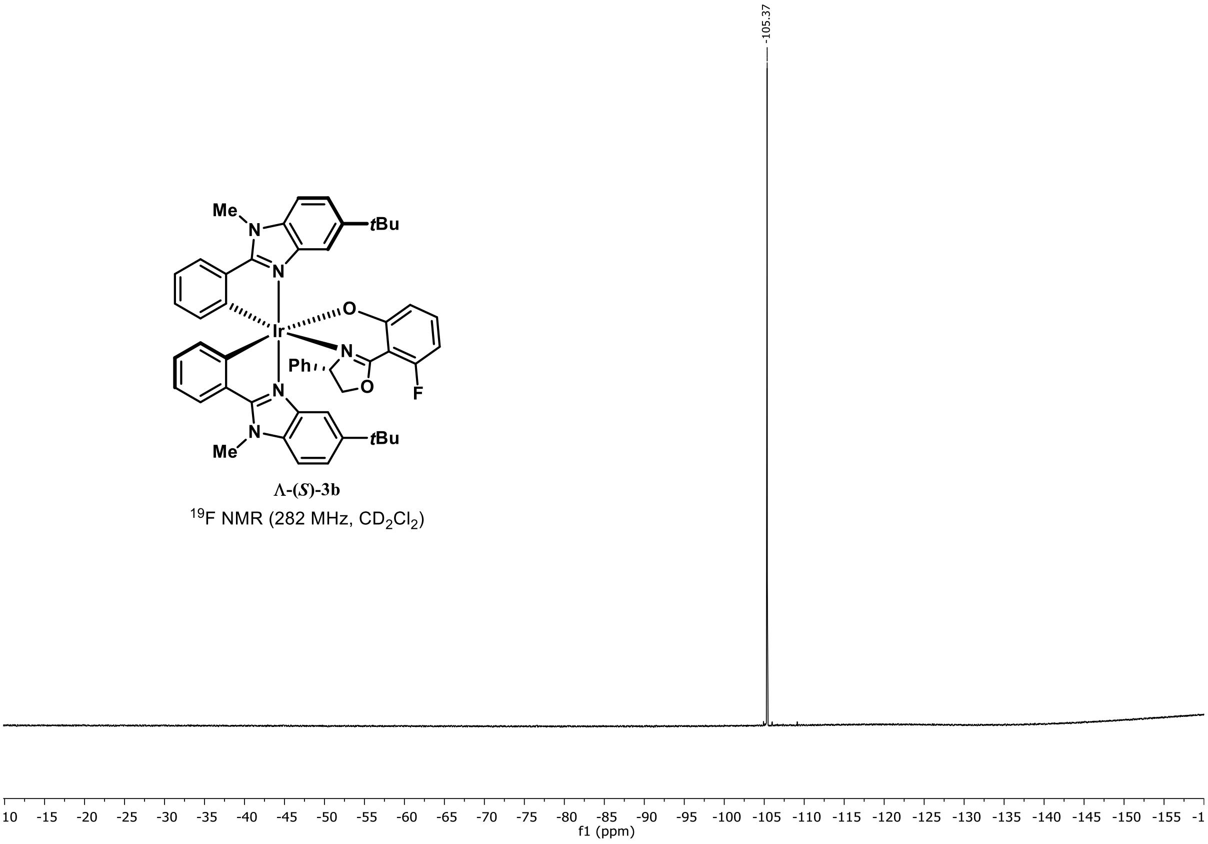

Figure S10: ^{19}F NMR Spectrum of Λ-(*S*)-**3b**.

Δ-(*S*)-3b

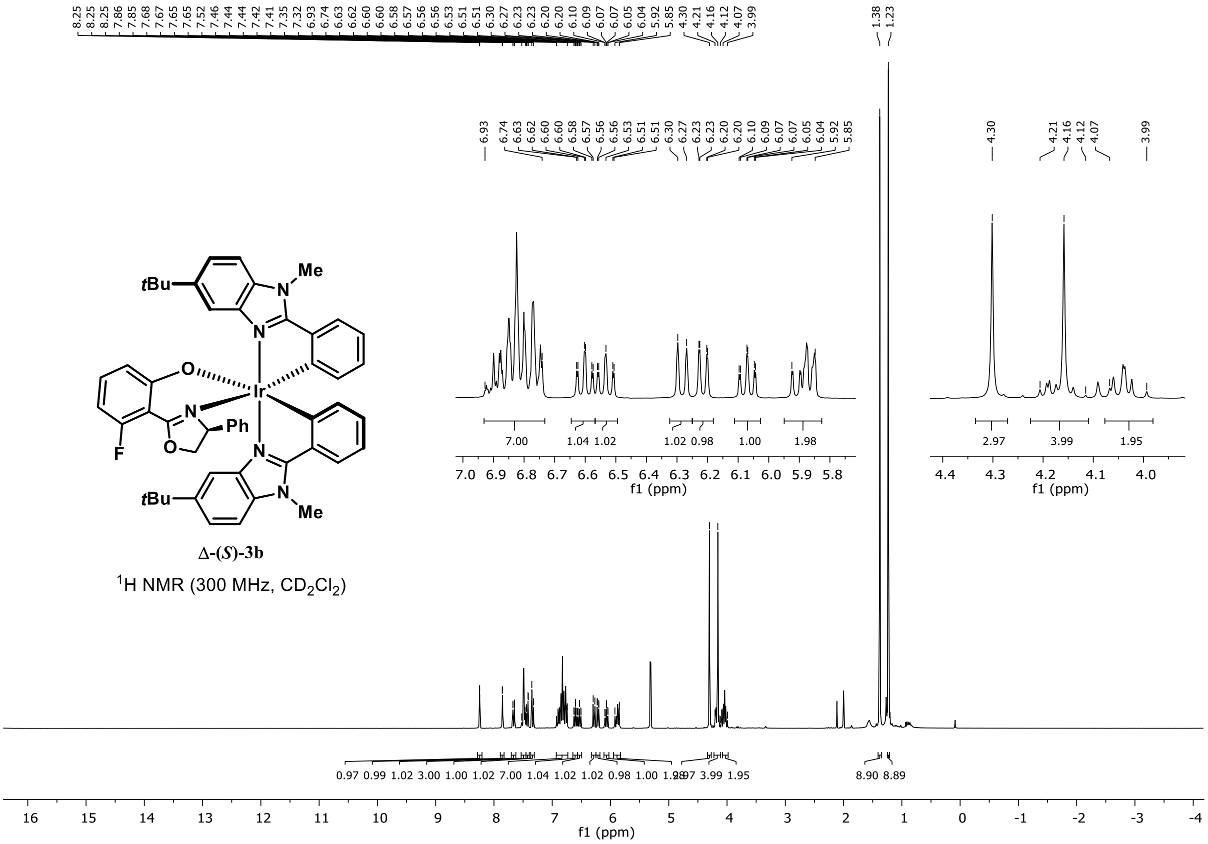

Figure S11: ^{1}H NMR Spectrum of Δ-(*S*)-**3b**.

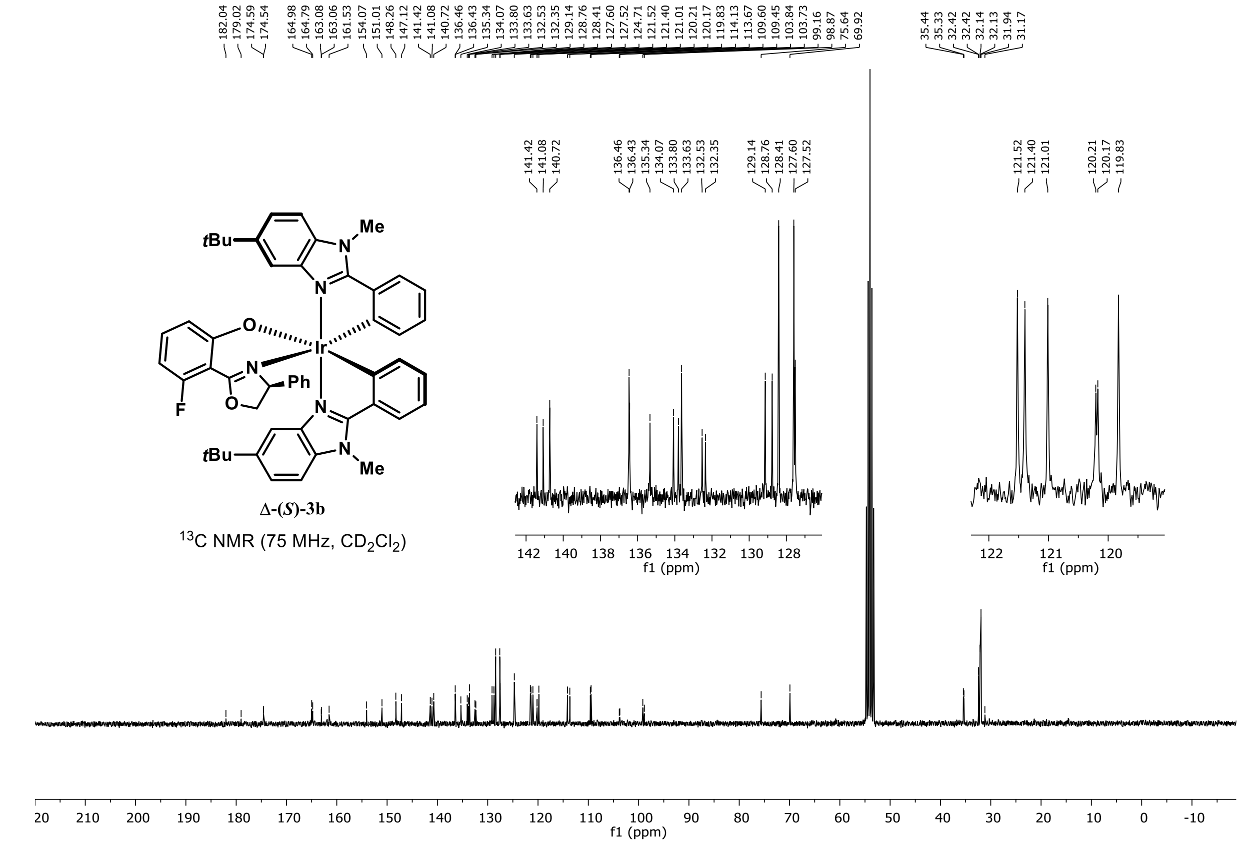

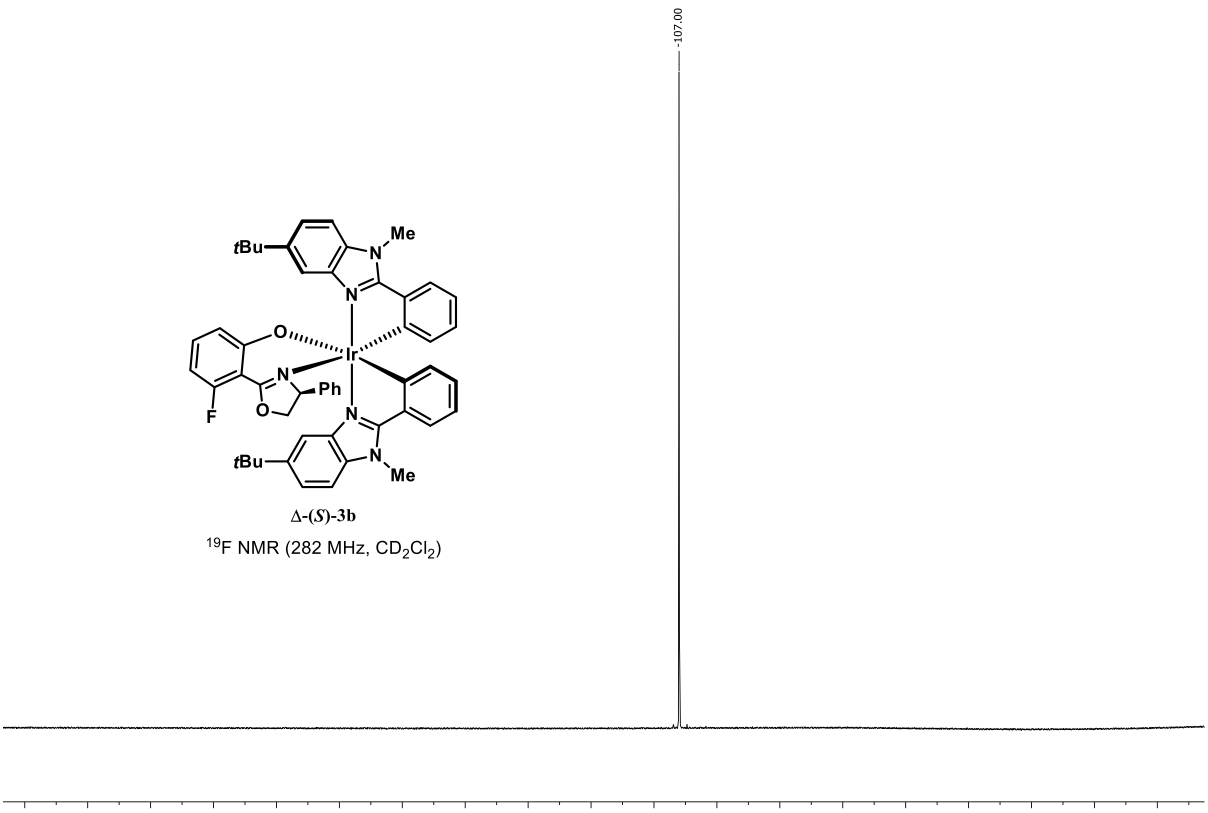

Figure S12: ^{13}C and ^{19}F NMR Spectra of Δ-(*S*)-**3b**.

Λ-**IrInd**

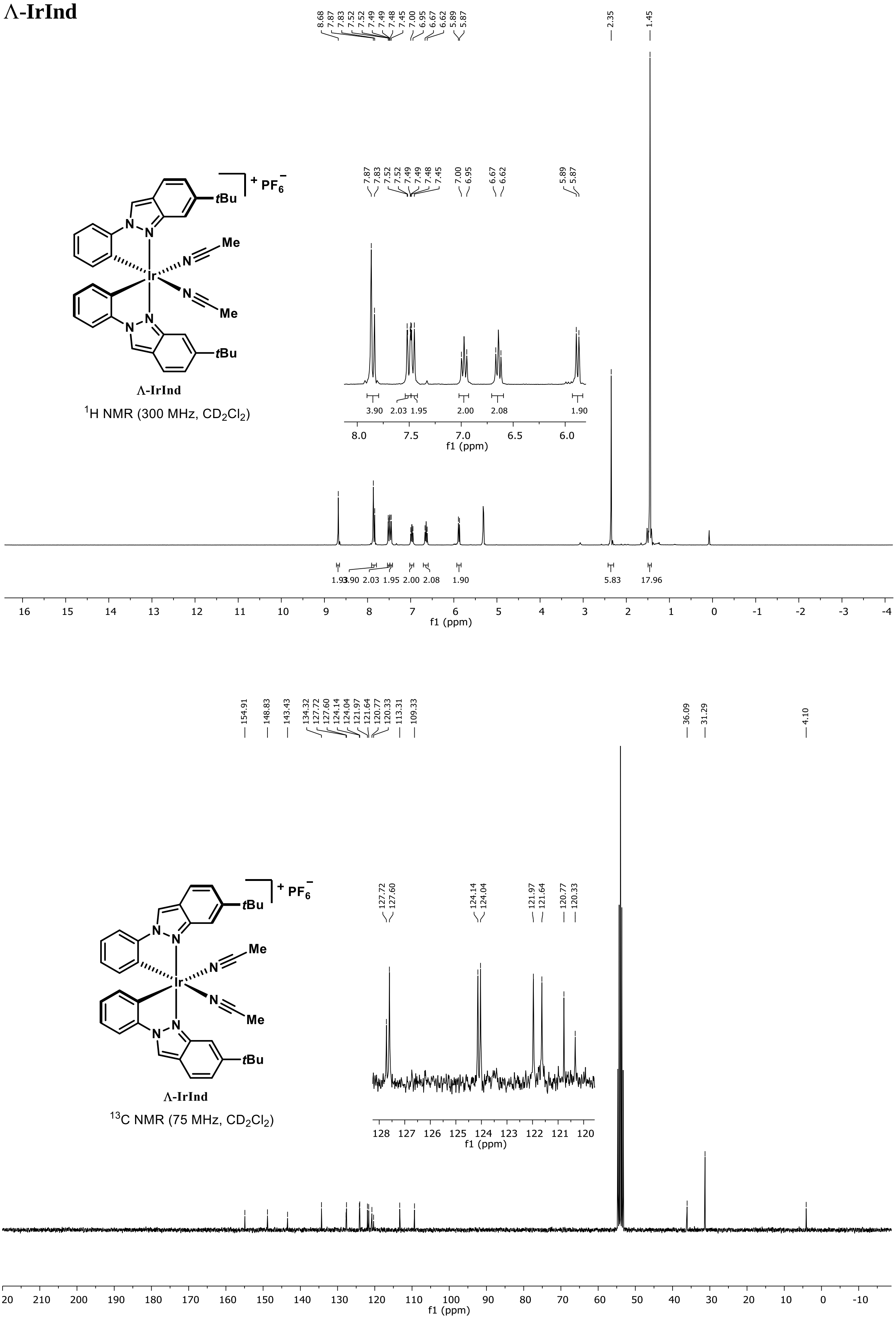

Figure S13: ^{1}H- and ^{13}C NMR Spectra of Λ-**IrInd**.

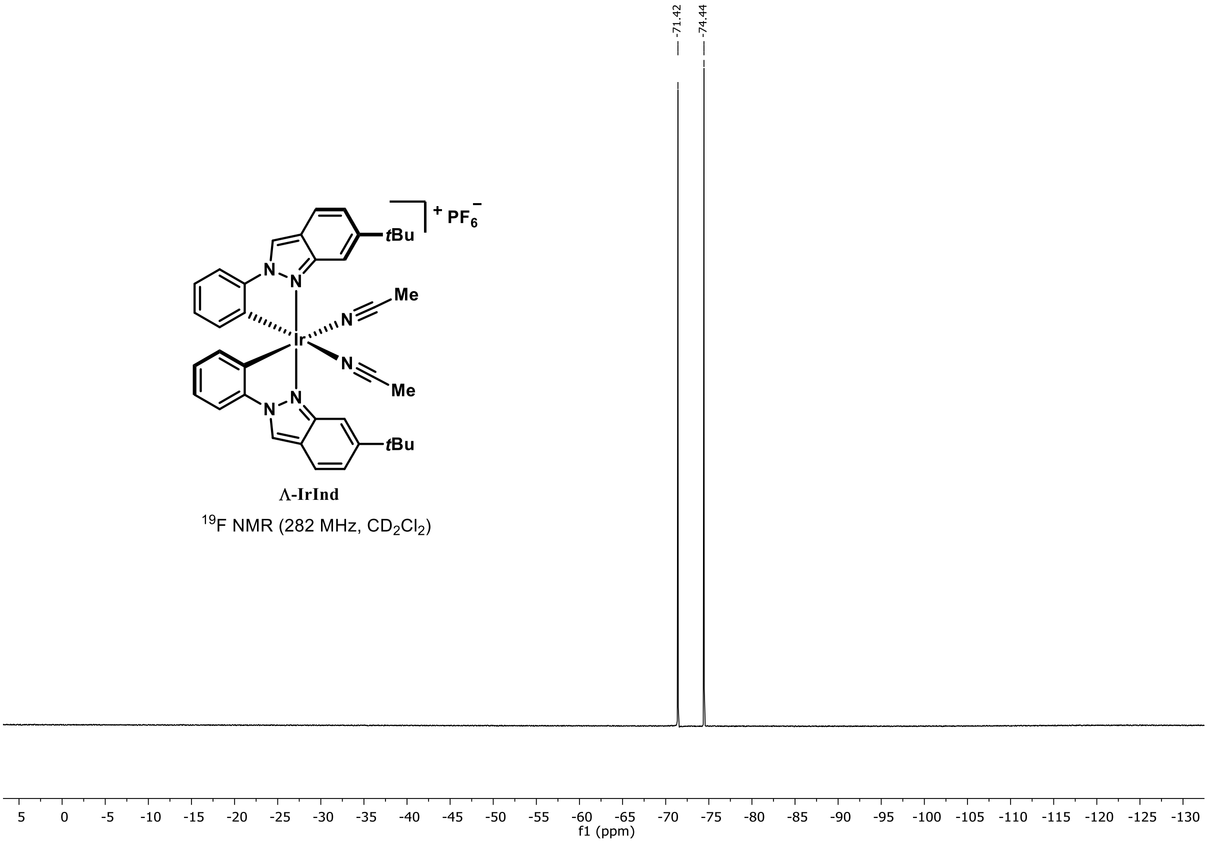

Figure S14: ^{19}F NMR Spectrum of Λ-**IrInd**.

The spectra of Δ-**IrInd** are identical and will not be shown.

Λ-IrBim

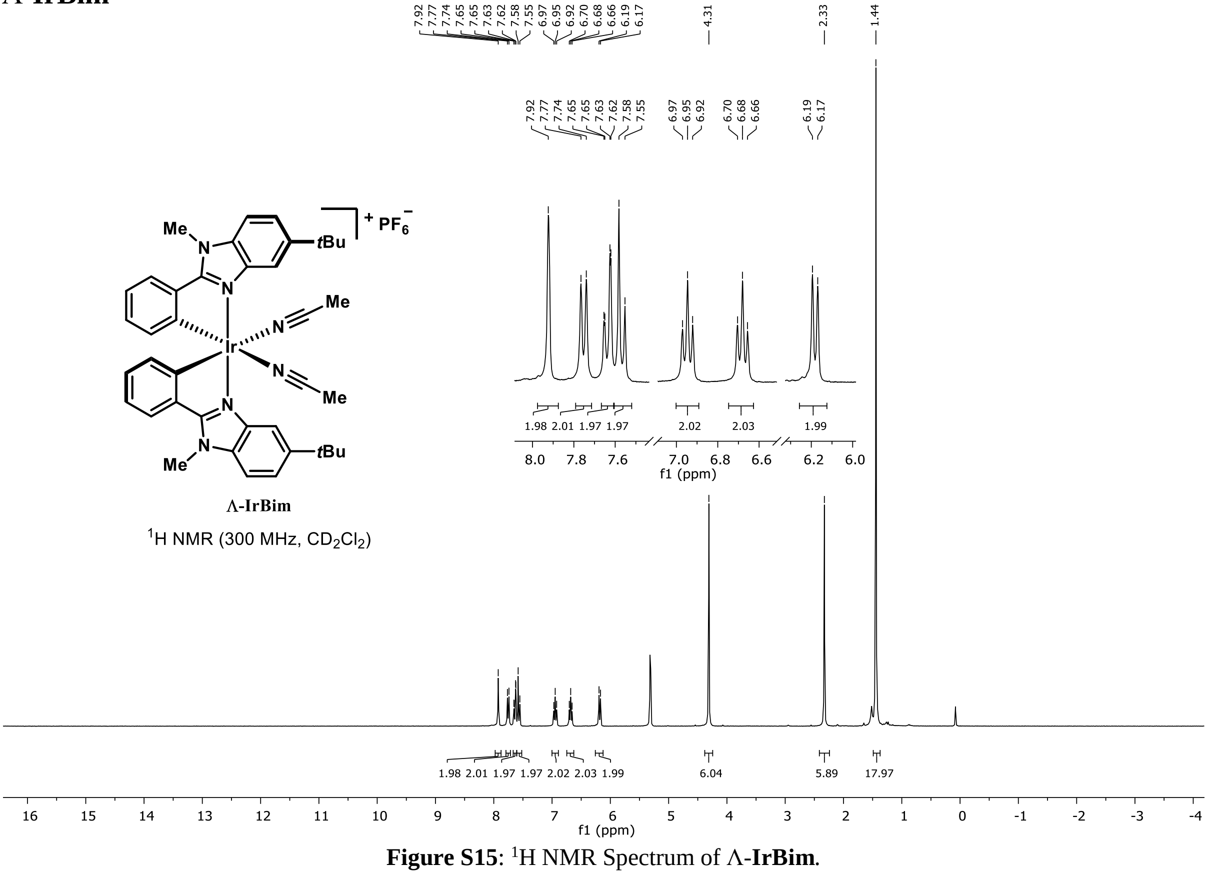

Figure S15: ^{1}H NMR Spectrum of Λ-**IrBim**.

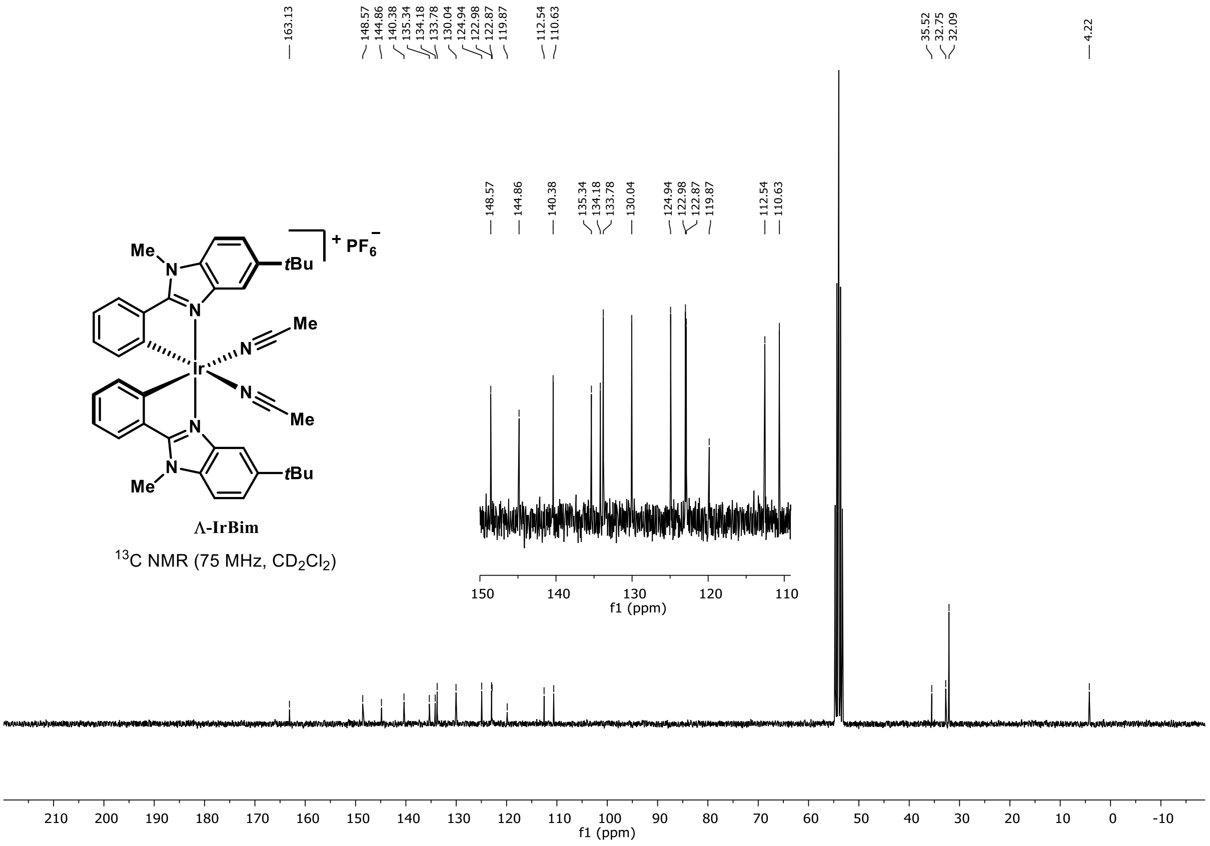

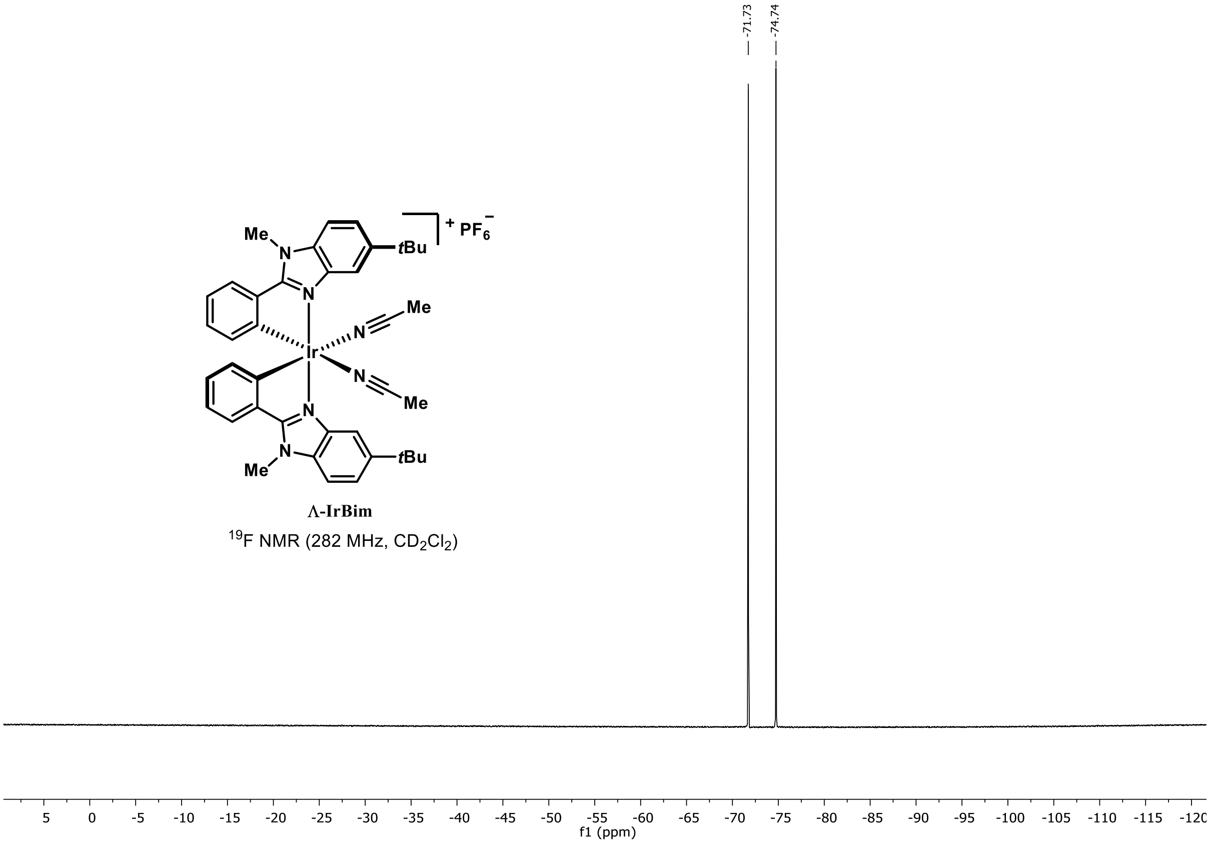

Figure S16: ^{13}C and ^{19}F NMR Spectrum of Λ-**IrBim**.

The spectra of Δ-**IrBim** are identical and will not be shown.

2. HPLC Traces

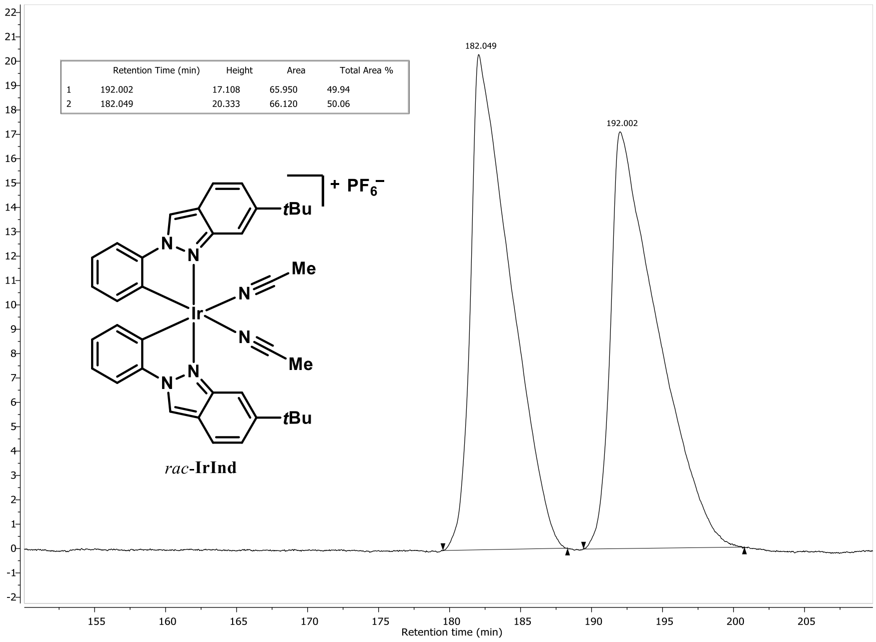

Figure S17: HPLC traces of *rac*-**IrInd**.

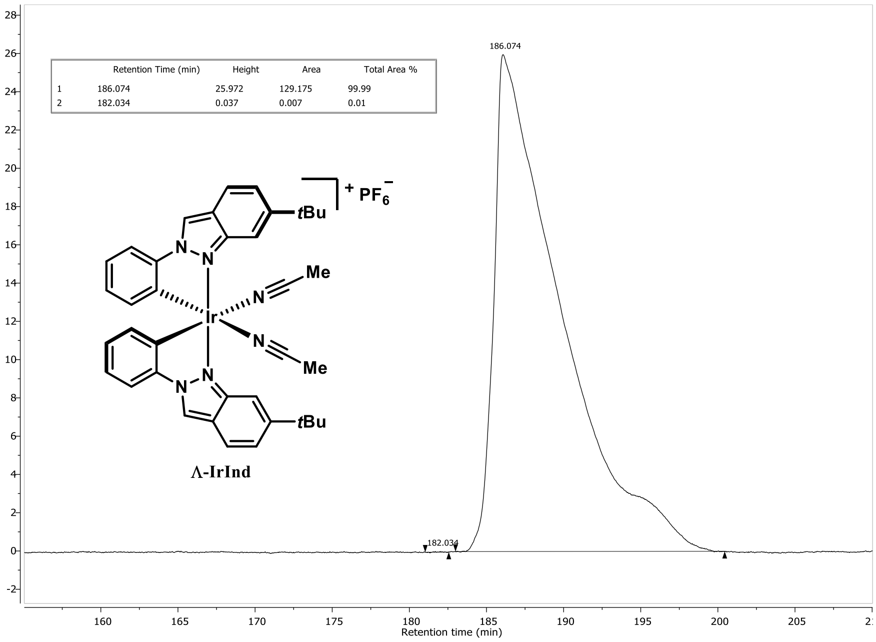

Figure S18: HPLC traces of Λ-**IrInd** (>99% ee).

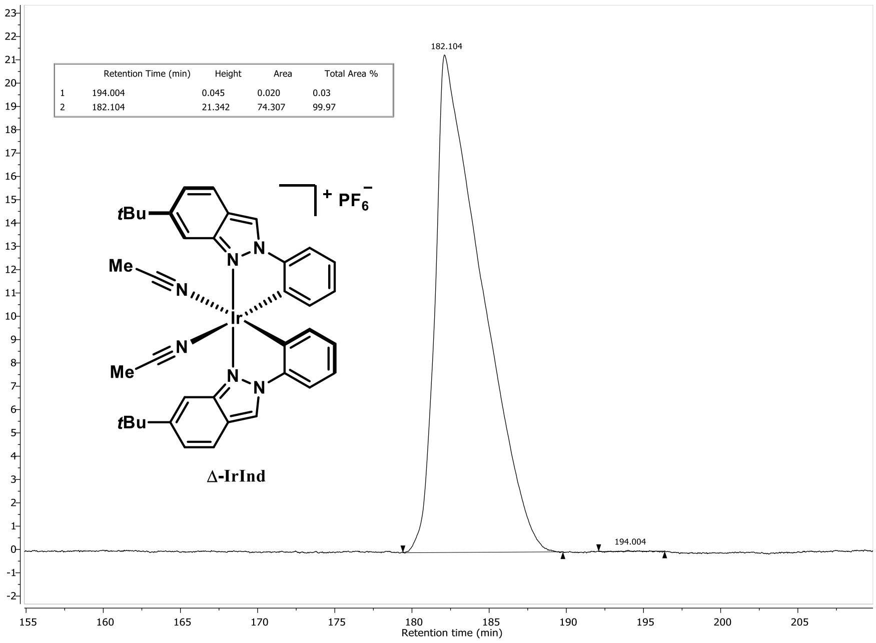

Figure S19: HPLC traces of Δ-**IrInd** (>99% ee).

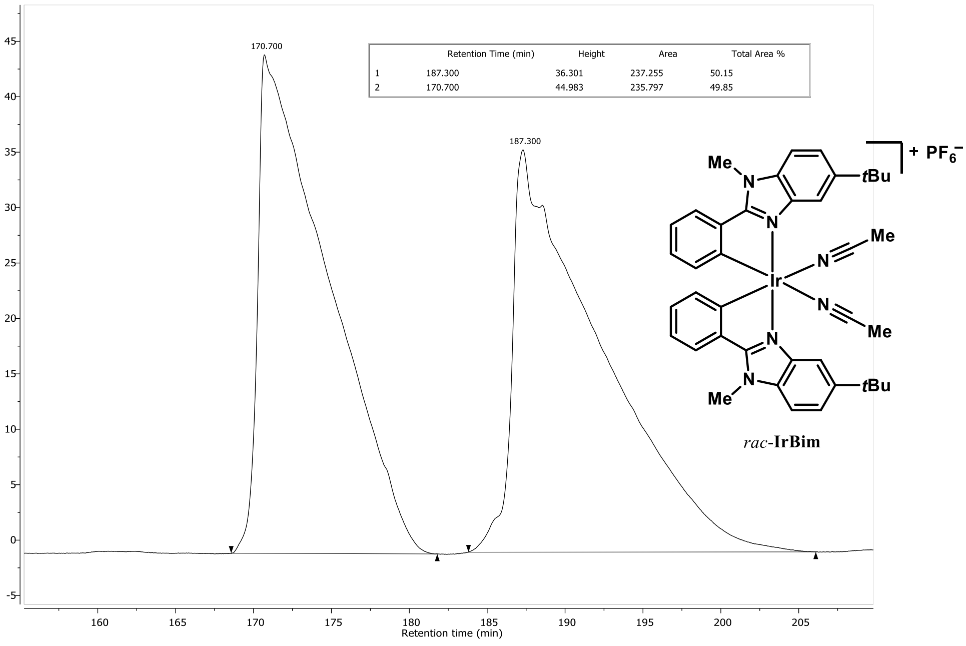

Figure S20: HPLC traces of *rac*-**IrBim**.

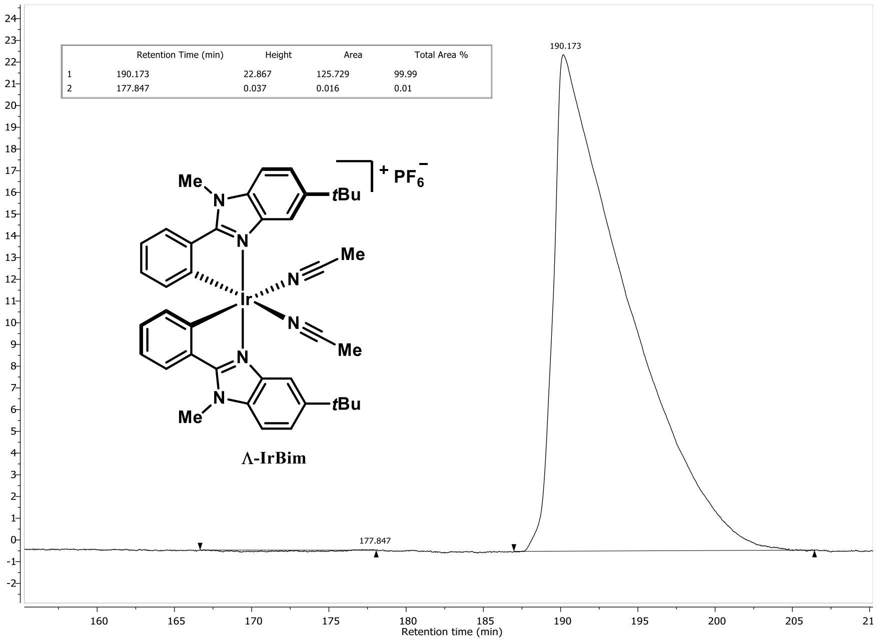

Figure S21: HPLC traces of Λ-**IrInd** (>99% ee).

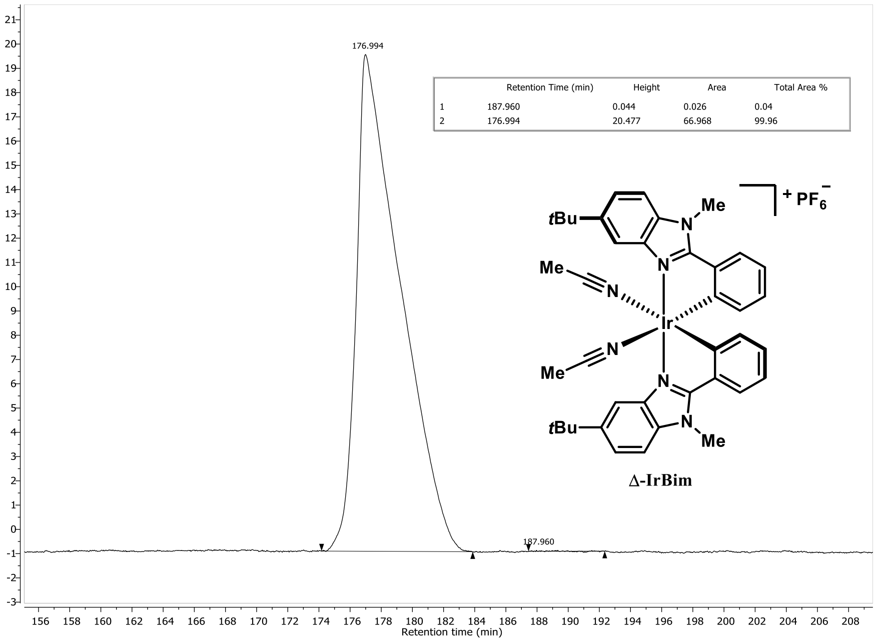

Figure S22: HPLC traces of Δ-**IrInd** (>99% ee).

3. Single Crystal X-Ray Diffraction

X-ray data were collected either with a STOE STADIVARI diffractometer or with a BRUKER D8 QUEST diffractometer.

Conditions using the STOE STADIVARI diffractometer:

Data was collected with a STOE STADIVARI diffractometer equipped with CuK_a radiation, a graded multilayer mirror monochromator (λ = 1.54178 Å) and a DECTRIS PILATUS 300K detector using an oil-coated shock-cooled crystal at 100(2) K. Absorption effects were corrected semi-empirical using multiscanned reflexions (STOE LANA, absorption correction by scaling of reflection intensities.). The number of observed reflections of the data collection used for cell constant refinement is pictured in table **S1** (cell determination). The structure was solved by direct methods by using the program XT V2014/1 (Bruker AXS Inc., 2014) and refined by full matrix least squares procedures on F^2 using SHELXL-2018/3 (Sheldrick, 2018). The non-hydrogen atoms have been refined anisotropically, carbon bonded hydrogen atoms were included at calculated positions and refined using the 'riding model' with isotropic temperature factors at 1.2 times (for CH_3 groups 1.5 times) that of the preceding carbon atom. CH_3 groups were allowed to rotate about the bond to their next atom to fit the electron density.

Conditions using the BRUKER D8 QUEST diffractometer:

Data was collected with a Bruker D8 QUEST area detector diffractometer equipped with MoK_α radiation, a graded multilayer mirror monochromator (λ = 0.71073 Å) and a PHOTON-100 CMOS detector using an oil-coated shock-cooled crystal at 100(2) K. Absorption effects were corrected semi-empirical using multiscanned reflexions (SADABS-2016/2 - Bruker AXS area detector scaling and absorption correction). The number of observed reflections of the data collection used for cell constant refinement is pictured in table **S1** (cell determination). The structure was solved by direct methods by using the program XT V2014/1 (Bruker AXS Inc., 2014) and refined by full matrix least squares procedures on F^2 using SHELXL-2018/3 (Sheldrick, 2018). The non-hydrogen atoms have been refined anisotropically, carbon bonded hydrogen atoms were included at calculated positions and refined using the 'riding model' with isotropic temperature factors at 1.2 times (for CH_3 groups 1.5 times) that of the preceding carbon atom. CH_3 groups were allowed to rotate around the bond to their next atom to fit the electron density.

Single crystals suitable for X-ray diffraction were prepared from a concentrated solution of the respective iridium(III)-complex (about 1.5 mg) in MeCN, which was transferred to a regular NMR tube. THF (about 0.1 mL) was added and the mixture obtained was carefully layerd with Et_2O (about 3.0 mL), the tube was sealed and the biphasic mixture was left standing at room temperature over night to allow the diffusion of layers. If no formation of suitable crystals was observed after that time, the NMR-tube

was laid down horizontally for another 12 h. Crystal structures, data and details of the structure determination are presented in figure **S27** and in table **S1**.

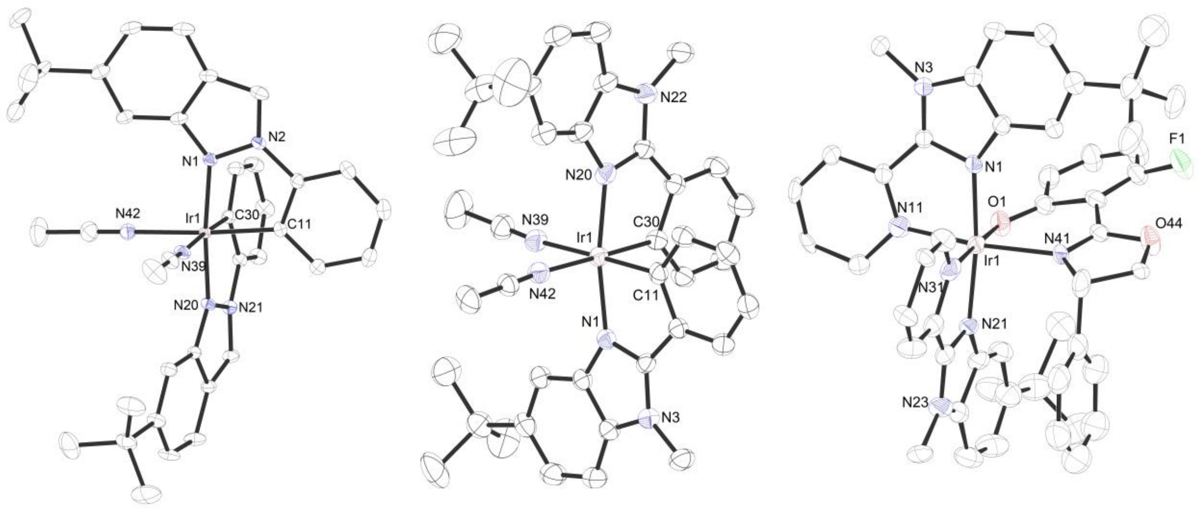

Figure S23: Crystal structures of Λ-**IrInd**, *rac*-**IrBim** and Λ-(*S*)-**3b** (from left to right). Solvent molecules, hydrogen atoms and PF_6-counterions are omitted for clarity. ORTEP drawing with 30% probability of thermal ellipsoids.

Table S1: Crystal data and details for structure determination.

	**Λ-IrInd**	*rac*-**IrBim**	Λ-(*S*)-**3b**
Idenfication code, Diffractometer	SBMM97C4 BRUKER D8 QUEST	SBMM92C2 STOE STADIVARI	SBMM94FA BRUKER D8 QUEST
Habitus, colour	nugget, green	nugget, yellow	prism, green
Empiric formula	$C_{38.50}H_{41.50}ClF_6IrN_6O_{0.25}P$	$C_{45}H_{51.50}F_6IrN_{8.50}P$	$C_{51}H_{53}Cl_4FIrN_7O_2$
Cell determination	9803 peaks with Theta 2.3 to 27.5°	63070 peaks with Theta 3.0 to 75.9°	1573 peaks with Theta 1.7 to 19.7°
Formula weight	964.89	1048.61	1149.00

Crystal system, space group	Monoclinic $P2_1$	Triclinic P-1	Tetragonal $P4_1$
a, *b*, *c* (Å)	a = 12.3670(7)<br>b = 25.9412(14)<br>c = 12.8263(8)	a = 13.2935(3)<br>b = 13.3338(3)<br>c = 16.0770(3)	a = 11.9791(8)<br>b = 11.9791(8)<br>c = 36.924(2)
α, β, γ (°)	α = 90<br>β = 104.9146(18)<br>γ = 90	α = 73.180(2)<br>β = 65.551(2)<br>γ = 65.046(2)	α = 90<br>β = 90<br>γ = 90
V (Å^3)	3975.4(4)	2329.15(10)	5298.5(8)
Z	4	2	4
μ (mm^{-1})	3.531	6.424	2.769
Crystal size (mm)	0.50 x 0.20 x 0.11	0.24 x 0.12 x 0.11	0.52 x 0.15 x 0.14
No. of measured, independent and observed [I>2σ(I)] reflections	304147<br>26035<br>25196	64659<br>9374<br>8228	48166<br>9822<br>8641
R(int)	0.0345	0.0410	0.0592
Goodness-of-fit on F^2	1.104	1.021	1.131
R index (all data)	wR2 = 0.0402	wR2 = 0.0840	wR2 = 0.1548
R index conventional [I>2σ(I)]	R1 = 0.0201	R1 = 0.0320	R1 = 0.0648

No. of reflections	26035	9374	9822
No. of parameters	1120	828	784
No. of restraints	157	966	1460
T_{max}, T_{min}	0.2154, 0.1332	0.1054, 0.0236	0.700, 0.320
$\Delta\rho_{max}$, $\Delta\rho_{min}$ (e Å^{3})	0.685, –1.258	1.367, –0.795	1.481, –2.042
Temperature (K)	100(2)	100(2)	100(2)
Wavelength (Å)	0.71073	1.54178	0.71073
Flack parameter (absolute structure)	–0.0077(9)	-	0.067(19)
